# Sequential Conformational Changes in the Morbillivirus Attachment Protein Initiate the Membrane Fusion Process

**DOI:** 10.1371/journal.ppat.1004880

**Published:** 2015-05-06

**Authors:** Nadine Ader-Ebert, Mojtaba Khosravi, Michael Herren, Mislay Avila, Lisa Alves, Fanny Bringolf, Claes Örvell, Johannes P. Langedijk, Andreas Zurbriggen, Richard K. Plemper, Philippe Plattet

**Affiliations:** 1 Division of Neurological Sciences, Department of Clinical Research and Veterinary Public Health (DCR-VPH), Vetsuisse Faculty, University of Bern, Bern, Switzerland; 2 Graduate School for Cellular and Biomedical Sciences, University of Bern, Bern, Switzerland; 3 Division of Laboratory Medicine, Karolinska University Hospital Huddinge, Stockholm, Sweden; 4 Janssen Infectious Diseases and Vaccines, Leiden, Netherlands; 5 Institute for Biomedical Sciences, Georgia State University, Atlanta, Georgia, United States of America; Icahn School of Medicine at Mount Sinai, UNITED STATES

## Abstract

Despite large vaccination campaigns, measles virus (MeV) and canine distemper virus (CDV) cause major morbidity and mortality in humans and animals, respectively. The MeV and CDV cell entry system relies on two interacting envelope glycoproteins: the attachment protein (H), consisting of stalk and head domains, co-operates with the fusion protein (F) to mediate membrane fusion. However, how receptor-binding by the H-protein leads to F-triggering is not fully understood. Here, we report that an anti-CDV-H monoclonal antibody (mAb-1347), which targets the linear H-stalk segment 126-133, potently inhibits membrane fusion without interfering with H receptor-binding or F-interaction. Rather, mAb-1347 blocked the F-triggering function of H-proteins regardless of the presence or absence of the head domains. Remarkably, mAb-1347 binding to headless CDV H, as well as standard and engineered bioactive stalk-elongated CDV H-constructs treated with cells expressing the SLAM receptor, was enhanced. Despite proper cell surface expression, fusion promotion by most H-stalk mutants harboring alanine substitutions in the 126-138 “spacer” section was substantially impaired, consistent with deficient receptor-induced mAb-1347 binding enhancement. However, a previously reported F-triggering defective H-I98A variant still exhibited the receptor-induced “head-stalk” rearrangement. Collectively, our data spotlight a distinct mechanism for morbillivirus membrane fusion activation: prior to receptor contact, at least one of the morbillivirus H-head domains interacts with the membrane-distal “spacer” domain in the H-stalk, leaving the F-binding site located further membrane-proximal in the stalk fully accessible. This “head-to-spacer” interaction conformationally stabilizes H in an auto-repressed state, which enables intracellular H-stalk/F engagement while preventing the inherent H-stalk’s bioactivity that may prematurely activate F. Receptor-contact disrupts the “head-to-spacer” interaction, which subsequently “unlocks” the stalk, allowing it to rearrange and trigger F. Overall, our study reveals essential mechanistic requirements governing the activation of the morbillivirus membrane fusion cascade and spotlights the H-stalk “spacer” microdomain as a possible drug target for antiviral therapy.

## Author Summary

With the ultimate aim to develop *pan*-morbillivirus fusion inhibitors, we here characterized a potent neutralizing monoclonal antibody. The antibody recognizes the ectodomain of the membrane-bound tetrameric attachment (H) protein, which together with the fusion protein and a host cell receptor executes plasma membrane fusion to deliver the viral genetic information into the cell. The H-ectodomain consists of a short F-binding/activating stalk region supporting receptor-binding head domains. Molecular characterization of the identified mAb epitope (which locates in the membrane-distal stalk module called “spacer”), enabled us to unravel two sequential conformational changes occurring in CDV H-tetramers that stand at the core of the molecular mechanism translating receptor binding to F-triggering. We additionally propose that both rearrangements are triggered upon receptor-induced “de-activation” of an auto-repressed state assumed by H prior to receptor binding. This locked state enables H/F interaction while preventing premature F-activation. Furthermore, although paramyxovirus attachment proteins may fold into very similar “pre-receptor-bound” conformational states, the presence of the “spacer” module in the stalk emerges as a key determinant leading to distinct mechanisms of membrane fusion triggering.

## Introduction

Measles virus (MeV) is a major human pathogen leading to more than 120,000 deaths per year [1]. The disease can be prevented by vaccination, and global eradication is considered feasible in principle, but requires maintenance of a 95% herd immunity. However, sub-optimal vaccine delivery in developing countries and non-compliance in western countries continue to foster measles outbreaks. In order to achieve global measles eradication, post-exposure prophylaxis has recently been proposed as a synergistic strategy to complement vaccination programs by filling herd immunity gaps [2] and newly available morbillivirus infection inhibitors have established proof-of-concept for the efficacy of this approach in animal models [3–5]. However, to minimize the possibility of emergence of drug-resistant mutants, development of additional candidate compounds for combined therapy is indicated.

MeV belongs to the *Morbillivirus* genus within the *Paramyxovirus* family, which also contains important animal pathogens such as canine distemper virus (CDV) or peste des petits ruminants virus (PPRV) [6]. CDV is one of the major infectious agents of carnivores and often induces severe neurological disorders [7]. Importantly, CDV exhibits a very broad host range that even extends to non-human primates [8–11], which raises concerns that the virus could eventually adapt to humans. Therefore, the development of a panel of broad-spectrum morbillivirus inhibitors might be important to augment measles eradication and suppress the emergence of future zoonotic morbilliviruses.

Both MeV and CDV entry systems rely on two surface glycoproteins for infection: the receptor-binding protein H and the fusion protein F [6]. Both proteins tightly associate to execute membrane fusion at neutral pH. It is assumed that H-protein binding to a specific cell surface receptor is translated into the triggering of the F-protein [12, 13]. Subsequently, F undergoes a series of irreversible conformational changes that lead to merger of the viral envelope with a host cell membrane, resulting eventually in the formation of a fusion pore [6, 14, 15].

Recent structural and biochemical studies revealed that tetramers represent the physiological oligomer of the morbillivirus H-protein [16, 17]. Each H-monomer contains a short luminal tail, a single transmembrane domain and a large ectodomain. The extracellular region is composed of a membrane-proximal stalk section supporting a membrane-distal cuboidal head domain with a six-beta propeller fold [16, 18–20], which is responsible for binding to multiple receptors (such as SLAM and Nectin-4) [18, 21–30]. The H-stalk is further divided into three modules: (i) a central section consisting of a candidate F-contacting segment (aa 110–118) [31, 32], which partially overlaps with an F-triggering region (aa 84–117), (ii) a compact intermediate “spacer” section (aa 122–137) with unknown function, and (iii) two C-terminal dimeric “linker” regions (aa 139–154) that may connect the four globular head domain to the stalks [33]. Although the precise structure of the morbillivirus H-stalk domain remains to be determined, the atomic structures of the related parainfluenza virus type 5 (PIV5) and Newcastle disease virus (NDV) attachment protein (HN)-stalks were partially resolved and revealed a conserved four-helical bundle (4HB) with an upper straight and lower supercoiled conformation [34–36]. Successful engineering of covalent bonds trapping dimers and/or tetramers throughout the CDV and MeV H-stalks indicate that the 4HB-like conformation is presumably a conserved theme among members of the *Paramyxovirus* family [31, 33, 37, 38].

The most recent model for triggering the paramyxovirus entry machinery is based on discrete crystal structures of soluble form of the PIV5 and NDV receptor-binding proteins (HN). In these structures, either one (PIV5-HN) or two (NDV-HN) dimeric head units backfold onto the C-terminal region of the stalk, thereby covering the putative F-activation/binding site (referred to as “heads down” conformation) [34, 36]. Alternative structures were also reported in which both dimeric head units are assembled into tetramers (referred to as “heads up” conformation) [39]. These static atomic structures inferred the possibility that receptor-binding may “shift” the dimeric head units from the “down” to the “up” configuration, hence unmasking the F-activation/binding site. In turn, the stalks are freed to mediate interaction with and activation of prefusion F-complexes (referred to as the “stalk-exposure” and “induced fit” models) [13, 34, 36, 40]. Overall, the models illustrate the essential role of the attachment protein’s “heads down” conformational state, which prevents F-binding prior to receptor engagement.

Several lines of evidence indicate that the “stalk-exposure” model may act as a general mechanism regulating paramyxovirus entry: (i) paramyxovirus attachment protein stalk domain is involved in short-range interaction with the F-protein [31, 32, 41–45], (ii) although unregulated, PIV5 HN “headless” constructs remain bioactive [46]; a phenotype that equally extends to MeV [47, 48], Nipah (NiV) [49], NDV and Mumps (MuV) attachment protein stalk regions [40], (iii) PIV5 HN and F do not assemble intracellularly [46, 50, 51], (iv) biochemical and structural data indicate that MeV H can adopt discrete conformations [16, 38] and (v) a polyclonal antibody generated against the stalk domain of the Nipah virus attachment protein (G) can sense a receptor-induced conformational change in the stalk [49].

However, several studies challenge that the “stalk-exposure” model: (i) the morbillivirus and henipavirus’ glycoproteins preassemble intracellularly [48, 52–56], (ii) stalk-elongated MeV H-mutants remain bioactive [57], (iii) only tetrameric MeV H-heads conformations (in complex with SLAM) were crystalized [16], and (iv) human parainfluenza type 3 (HPIV3) glycoproteins may also associate prior to receptor-binding [58, 59], where the attachment protein (HN) would exert a stabilizing role on prefusion F-structures [60]. These findings suggest that the entry system of these paramyxoviruses either does not rely on the “stalk-exposure” model or that variations of the latter must exist.

To elucidate the morbillivirus F-triggering machinery, we have characterized a monoclonal antibody (mAb-1347) that very efficiently ablated morbillivirus-mediated virus-to-cell and cell-to-cell fusion activities. Molecular mapping of the mAb-1347 epitope revealed that the H-stalk C-terminal “spacer” region contains the putative antibody-docking site. Strikingly, mAb-1347 spotlighted a yet unrecognized receptor-induced “head-stalk” conformational change in morbillivirus H, which occurs prior to the “opening” of the central stalk section required for F-activation. Furthermore, alanine-scanning mutagenesis highlighted a key functional role of the “spacer” microdomain in enabling H-tetramers to fold into a putative critical pre-receptor-bound state. These findings are discussed in the context of a further refined model of morbillivirus membrane fusion triggering, which illustrates how paramyxoviruses may have evolved diverging mechanisms to initiate the F-protein refolding cascade necessary for cell entry.

## Results

### Anti-CDV-H mAb-1347 inhibits both viral-cell and cell-to-cell fusion without influencing H/SLAM binding activity

**Fig 1:**
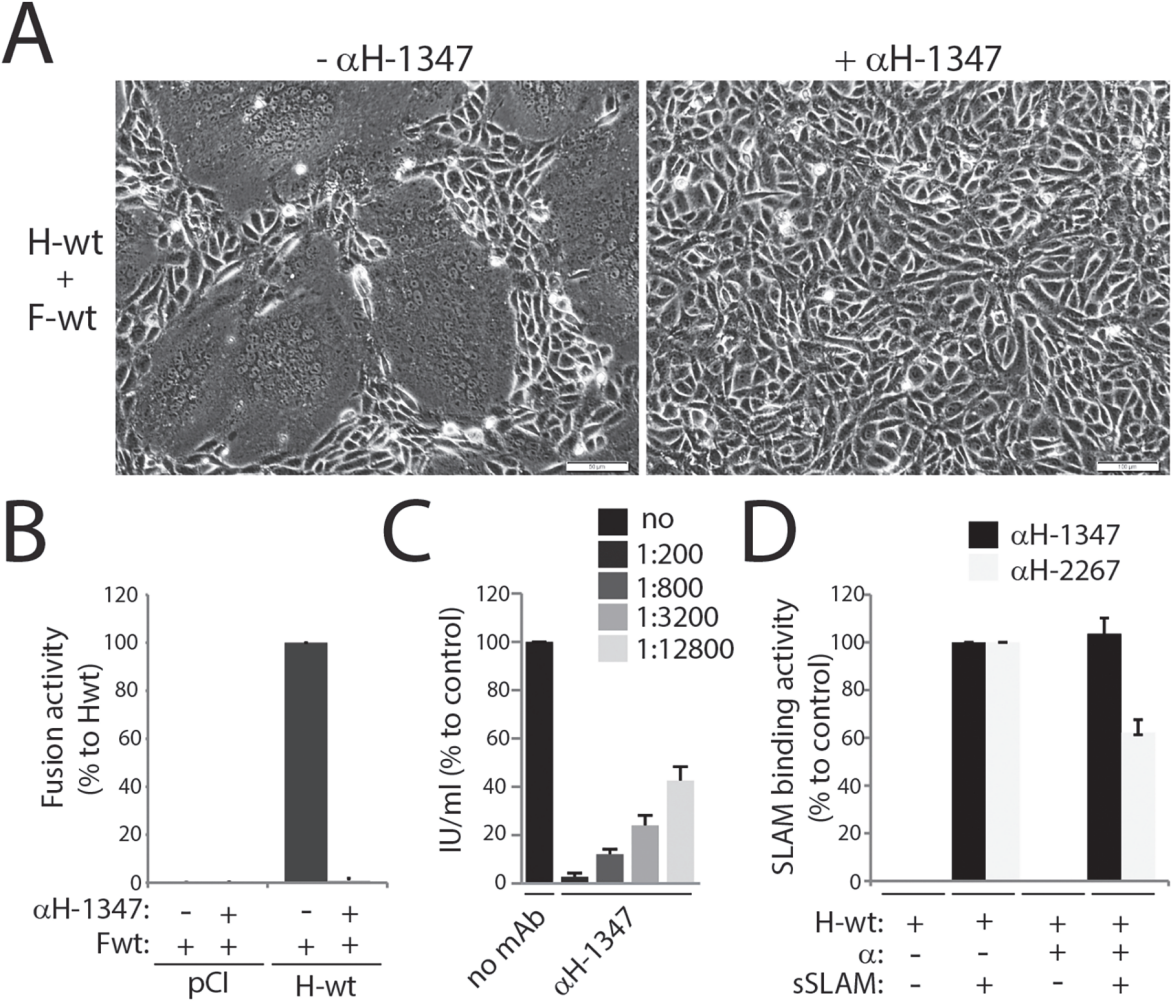
Inhibition of CDV-mediated viral-cell and cell-cell fusion by the anti-CDV-H mAb-1347. (A) Syncytium formation assay. Cell-to-cell fusion activity in Vero-cSLAM cells triggered by co-expression of CDV H-wt and CDV F-wt (A75/17 strain) in the presence of absence of mAb αH-1347. Representative fields of view of cell-cell fusion induced 24h post-transfection are shown. (B) Quantitative fusion assay. Vero-cSLAM cells (target cells) were infected with MVA-T7 (MOI of 1). In parallel, a population of Vero cells (effector cells) was transfected with the F-wt and H-wt-expressing vectors and a plasmid containing the luciferase reporter gene under the control of the T7 promoter. Twelve hours after transfection, effector cells were mixed with target cells and seeded into fresh plates. After 2.5 h at 37°C, fusion was indirectly quantified by using a commercial luciferase-measuring kit. For each experiment, the value obtained for the standard F/H combination was set to 100%. (C) Virus neutralization assay. A total of 100 infectious units of recA75/17^red^ was incubated with the indicated dilution of antibody for 1 h at 37°C. The virus-antibody mixtures were then added to 3h on Vero cells, overlaid with agar-containing medium and further incubated for 72 h at 37°C. Cell entry efficiency was determined by counting the number of red fluorescent syncytia induced by recA75/17^red^. (D) Effect of mAbs on H/SLAM binding efficiency. Vero cells were transfected with H-wt. Prior to treatment with soluble HA-tagged cSLAM molecules, mAbs were added as indicated, and SLAM-binding activity was calculated as the ratio of mean fluorescence intensities obtained with an anti-HA polyclonal Ab values (staining for sol. cSLAM) normalized to the levels obtained with the anti-FLAG mAb (staining for H). Values recorded for H-wt/cSLAM-binding efficiency in the absence of the mAb were set at 100%. Wt: wild type, α: monoclonal antibody, sSLAM: soluble version of cSLAM. Means ± S.D. of data from three independent experiments in triplicate are shown.

To better understand the mechanism of morbillivirus F triggering, we characterized a panel of monoclonal antibodies that was previously raised against CDV [61]. We first classified these mAbs to identify those that are most efficient in blocking both CDV-mediated cell entry (virus-to-cell fusion) and spread (cell-to-cell fusion). While virus-to-cell fusion inhibition was investigated by virus neutralization assays, block of cell-to-cell fusion was assessed by determining the capacity of the different mAbs to efficiently ablate H/F-mediated fusion of receptor-positive Vero-cSLAM cells. Our screen demonstrated that the anti-CDV-H monoclonal antibody 1347 (mAb-1347) strongly inhibited both types of fusion (Fig 1A–1C). To further investigate whether fusion inhibition correlated with prevention of receptor binding, a previously developed semi-quantitative SLAM-binding assay was employed. Results shown in Fig 1D illustrate that mAb-1347 did not alter SLAM binding activity, whereas the anti-CDV-H mAb-2267 used for comparison impaired H-SLAM binding by approximately 40%.

These data demonstrate that the anti-CDV-H monoclonal antibody 1347 effectively inhibits viral cell entry and spread by a mechanism other than blocking receptor binding.

### Epitope mapping of mAb-1347 revealed section 126–133 of the H-stalk as the putative antibody-docking site

**Fig 2:**
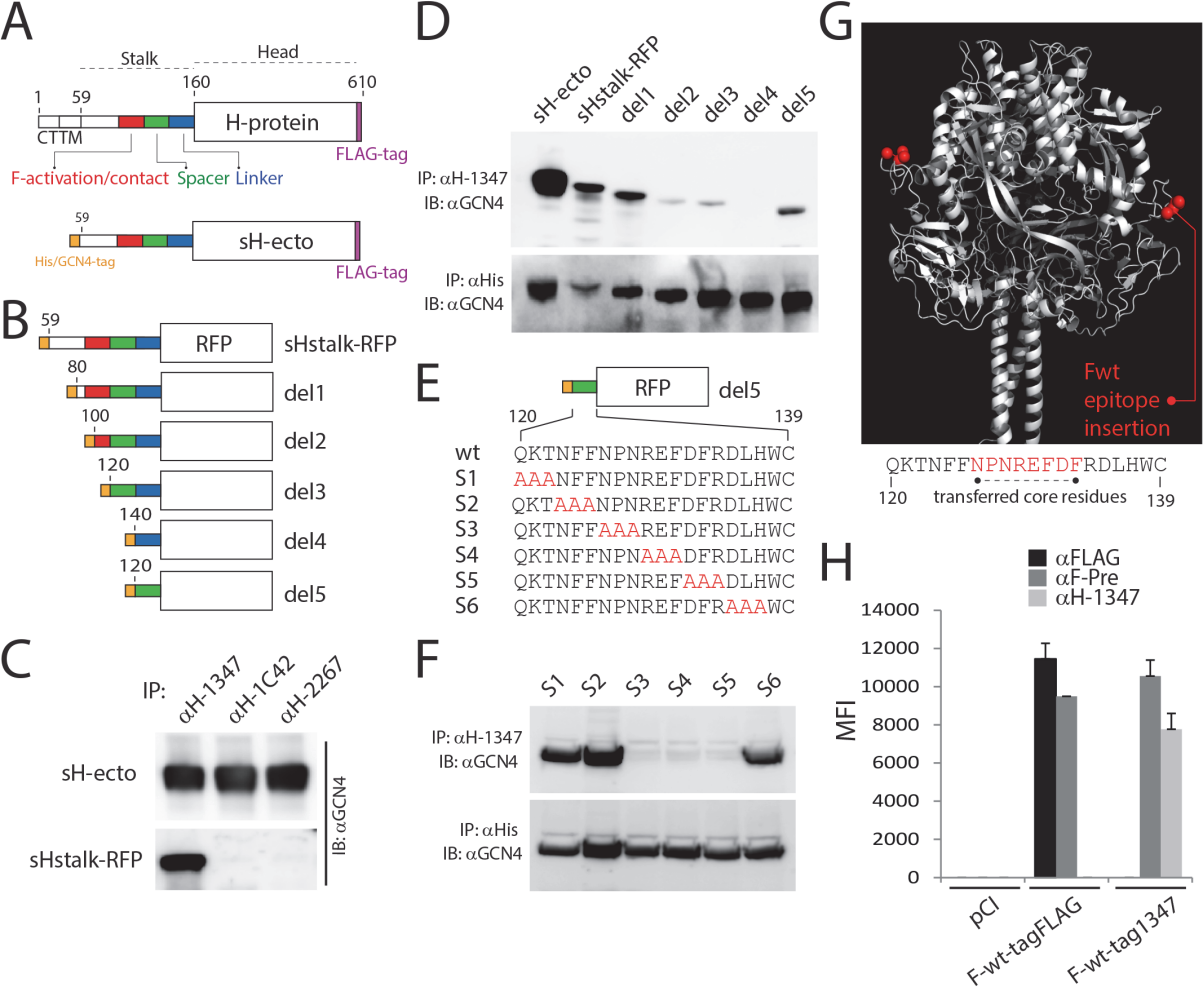
mAb αH-1347 epitope mapping. (A) Schematic representation of the full length CDV-H-protein (H-wt) and a derivative soluble form (sH-ecto). The main functional regions of the stalk are color-coded. CT: cytosolic tail; TM: transmembrane domain. (B) Schematic representation of soluble Hstalk-RFP chimeric proteins and derivative deleted mutants (1–5). Del: deletion; RFP: red fluorescent protein. Drawings are not to scale. (C, D and F) Immunoprecipitation experiments performed with the indicated mAb of soluble H forms derived from the supernatant of transfected 293T cells. Antigenic materials were detected using polyclonal anti-GCN4 (upper panel) or anti-His antibody (lower panel). IP: immunoprecipitation; IB: immunoblotting. (E) Upper part: Schematic representation of the deletion 5 soluble Hstalk-RFP. Lower part: alignment of the primary amino acid sequence of the CDV H-stalk “spacer” section and the derivative triple alanine-scan mutants. Alanines are highlighted in red. (G) Homology structural model of CDV-F and positions (in red) where the mAb αH-1347 epitope has been inserted. Lower part: primary amino acid sequence of the CDV H-stalk “spacer” section with the encompassed F-transferred mAb-1347 peptide (highlighted in red). (H) Reactivities of F-wt-tagFLAG or F-wt-tag1347 with the conformation-insensitive anti-FLAG, the F prefusion state-specific anti-F-Pre (4941) [61, 63] or the anti-CDV-H 1347 mAbs, probed 24 hours post-transfection in receptor-negative Vero cells. After addition of the secondary antibody, MFI values were recorded by flow cytometry analyses. Means ± S.D. of data from three independent experiments in triplicate are shown.

To further characterize the mechanism of mAb-1347 fusion inhibition, we attempted to identify its binding site on CDV H. Towards this goal, we employed a soluble H-form (sH-ecto; containing the full ectodomain and carrying a GCN4 motif fused N-terminally) [31]. In addition, we constructed a soluble protein consisting of the H-stalk that was flanked by GCN4 (N-terminally) and RFP (C-terminally). The whole cassette was cloned in frame with the IgK signal peptide to engineer a secreted version of the chimeric protein (sH-stalk-RFP). Both soluble H-constructs also carry a supplementary N-terminal hexahistidine tag (Fig 2A and 2B).

To determine whether H-stalks or H-heads were recognized by mAb-1347, both soluble proteins were expressed in 293T cells and culture supernatants subsequently incubated with different anti-CDV-H mAbs (1347, 1C42 and 2267). Immunoprecipitated proteins were subjected to western blot analysis and the results compared to the H-antigenic materials detected by a polyclonal anti-GCN4 antibody. Our data revealed that mAbs 1C42 and 2267 efficiently interacted with sH-ecto but not with sH-stalk-RFP, whereas mAb-1347 immunoprecipitated both soluble constructs (Fig 2C). Furthermore, when all four mAbs were tested for their reactivity against H-proteins gel fractionated under denaturing and reducing conditions, only mAb-1347 detected the H-antigenic material (S1A Fig). These data inferred that mAbs 1C42 and 2267 recognize conformational epitopes located in the H head domain, whereas mAb-1347 binds to a linear epitope located in the H-stalk section.

To further refine the region within the H-stalk recognized by mAb-1347, deletion mutants were generated based on the sH-stalk construct (Fig 2B). Whereas all soluble H-stalk mutants were efficiently immunoprecipitated by an anti-histidine epitope mAb, mAb-1347 completely lost reactivity with deletion mutant 4 (del4) (Fig 2D). Since the del4 mutant lacked only 20 residues (H-stalk section 120–139) compared to the del3 mutant, we engineered an additional construct containing only these 20 residues (del5). When subjected to pull-down analysis, del5 was efficiently immunoprecipitated by mAb-1347 (Fig 2D). Furthermore, when residues 120–122, 123–125 or 135–137 of del5 were substituted for alanines (Fig 2E), the resulting H-stalk mutants S1, S2 and S6 (scans 1, 2 and 6, respectively) were still recognized by the mAb, in contrast to S3, S4 and S5 (scans 3 to 5 spanning the 126–134 H-stalk region) (Fig 2F). Lastly, we transferred the eight core residues (126–133) of this segment into a location in the CDV F globular head domain that we have previously demonstrated to tolerate incorporation of short epitope-tags without substantially altering F bioactivity (F-wt-tagFLAG, Fig 2G). The resulting F-variant (F-wt-tag1347) was recognized by mAb-1347 as efficiently as by a control mAb (4941), which detects the prefusion conformation of the CDV F-trimer (Fig 2H).

To confirm these data in the context of membrane anchored H-protein, we expressed an H construct that lacked the head domains (headless CDV H; residues 1–159), which were replaced by FLAG epitope tags (S1B Fig). Headless CDV H was very efficiently recognized by mAb-1347, as demonstrated by immunofluorescence (IF) staining followed by flow cytometry (S1C Fig). Remarkably, and in contrast to H-wt, MFI values obtained with mAb-1347 and headless H were very similar to those recorded with the αFLAG mAb. Several truncated membrane-anchored headless CDV H versions were next engineered (all containing the FLAG tags) (S1B Fig). While mAb-1347 interacted efficiently with H stalk 1–159 and 1–139, a slightly decreased binding activity was recorded for headless 1–132 when compared to surface expression of this variant (monitored with an anti-FLAG mAb). Consistent with the results of our initial epitope mapping, mAb-1347 binding to headless H 1–122 was completely lost, despite efficient surface expression of this mutant (S1C Fig).

Taken together, our data identified residues 126–133, located in the C-terminal part of the H-stalk domain, as the critical amino acids governing mAb-1347 binding. Moreover, surface-exposed H-tetramers lacking the head domains interact more efficiently with mAb-1347 than standard H.

### mAb-1347 blocks spontaneous F-triggering by headless CDV H

**Fig 3:**
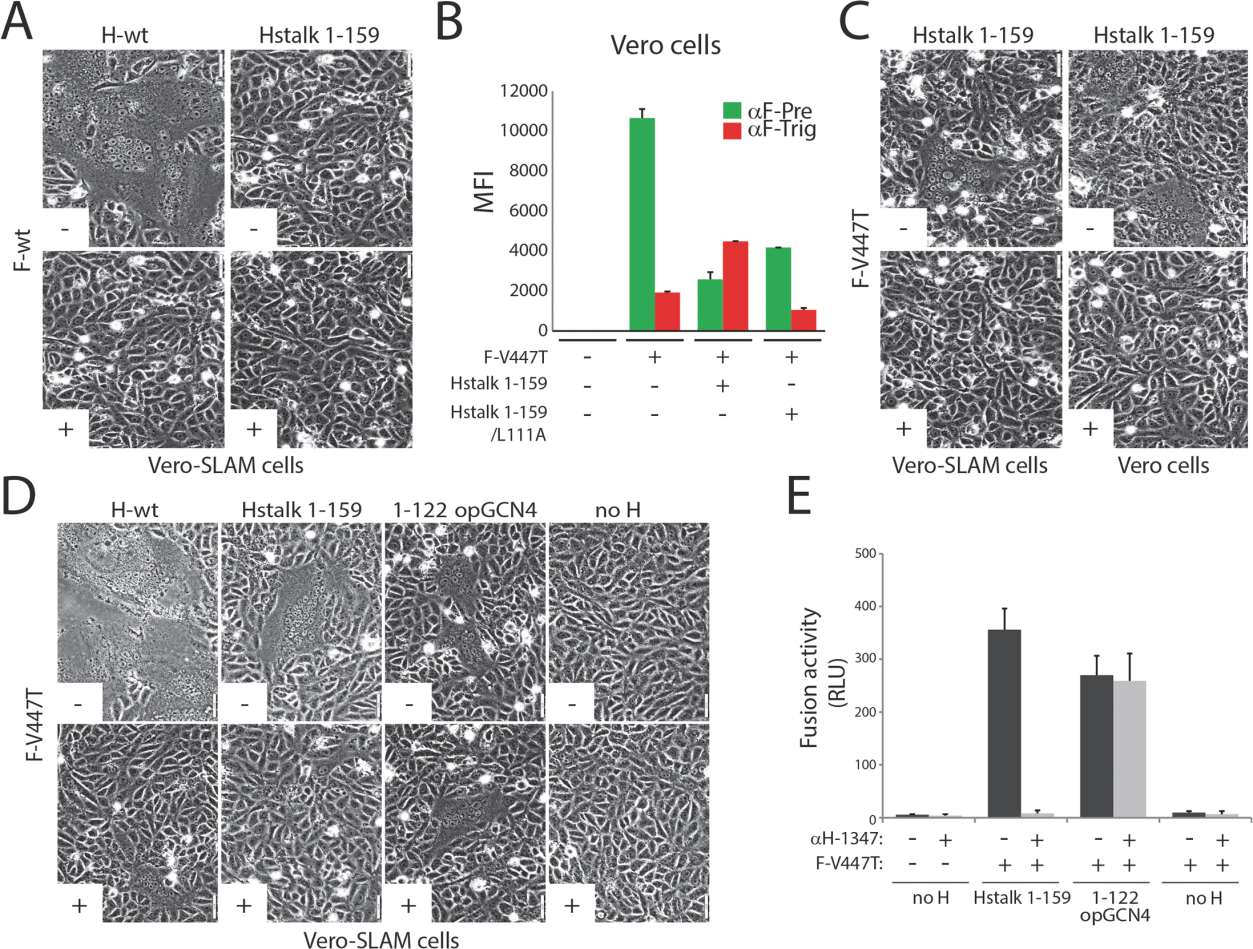
Inhibition of headless H/F-mediated membrane fusion by mAb αH-1347. (A, C and D) Syncytium formation assay. Cell-to-cell fusion activity in Vero or Vero-cSLAM cells triggered by co-expression of CDV H-wt or headless H variants with CDV F-wt or CDV F-V447T (A75/17 strain) [62] in the presence (+) of absence of mAb αH-1347 (-). Representative fields of view of cell-cell fusion induced 24h post-transfection are shown. (B) F-triggering assay. The CDV F-V447T mutant was expressed in Vero cells together with the indicated Hstalk variants. One day post-transfection, the conformation of the FLAG-tagged F-V447T mutant was monitored by probing its reactivity with an anti-F prefusion-specific mAb (4941; green histograms), triggered-specific mAb (3633; red histograms) [61, 63]. Secondary antibodies were added at 4°C, and to record quantitative values, mean fluorescence intensities were monitored by flow cytometry. (E) Quantitative fusion assays were performed as described in the legend of Fig 1B in the presence (light grey histograms) or absence (dark grey histograms) of mAb αH-1347. Means ± S.D. of data from three independent experiments performed in duplicates are shown.

It was recently reported that headless attachment protein constructs of other paramyxoviruses (PIV5, MeV, NiV, MuV and NDV) spontaneously trigger F-trimer refolding and membrane fusion, albeit to various extents [40, 46, 47, 49]. We therefore anticipated that headless CDV H may likewise trigger CDV F. Nevertheless, our initial attempts with wt CDV F remained unsuccessful, despite proper oligomerization and surface expression (Figs 3A and S1C and S2A–S2C). However, we recently demonstrated that prefusion F-trimers derived from the neurovirulent CDV A75/17 strain are conformationally highly stable [62] and identified a single substitution (V447T) that substantially destabilizes wt CDV F complexes to a level very similar to that of MeV F Edmonston. Using a previously established F-triggering assay [63], co-expression of CDV H stalk 1–159 with F-V447T led to efficient F activation (Fig 3B). Conversely, F-triggering was not induced by H stalk 1–159 with an additional L111A substitution (Fig 3B), which reportedly impairs F binding [32, 47]. To determine whether membrane fusion can be mediated by headless CDV H, we co-expressed Hstalk 1–159 and F-V447T in receptor-positive and-negative cells (Vero-SLAM and Vero cells, respectively) and assessed fusion qualitatively. Regardless of the cell line used, syncytia were detected, albeit at a low level (Fig 3C). Of note, fusion activity induced by CDV H stalk 1–159 remained below that observed for the heterologous MeV system. Interestingly, MeV H-stalk constructs required complex engineering to achieve proper stabilization and fusion promotion [47]. Hence, it is possible that headless CDV H naturally folds into a more stable conformation that enables fusion triggering, but tighter oligomerization subsequently interferes with optimal bioactivity.

Using the fusion-competent headless H/F-V447T combination, we asked whether mAb-1347 would still be capable of blocking F-triggering. Because this glycoprotein combination induced only very limited cell-to-cell fusion, we investigated fusion promoted by H stalk 1–159 qualitatively and quantitatively. Remarkably, headless H/F-mediated membrane fusion was completely inhibited by the mAb, whereas fusion proceeded unperturbed when F was triggered by an even shorter H 1–122 construct, regardless of the presence or absence of mAb-1347 (Fig 3D and 3E). Of note, CDV H-stalk 1–122 also required engineering of a stabilizing domain to achieve productive fusion triggering (1–122 opGCN4), analogous to the previous experience with headless MeV H proteins [47]. Overall, these data confirm that headless H 1–122 lacked the epitope recognized by mAb-1347, and illustrate that fusion inhibition by the antibody is independent of the presence of the H-head domains.

### Substitution of residues 128 and 131 in the MeV H-stalk increases the binding efficiency of mAb-1347

We next aligned the amino acid sequence of the CDV H-stalk 126–133 microdomain with those of several other prototypic canine distemper and morbillivirus strains. In agreement with a previously generated phylogenetic tree of morbillivirus H proteins, this alignment showed very good conservation between the selected CDV and PDV strains, whereas divergence was noticeable when compared to other morbillivirus H sequences (S3A Fig). As expected based on this observation, mAb-1347 likewise blocked fusion of other CDV strains (S3B Fig).

To determine whether mAb-1347 also blocks the related MeV H protein that contains two point mutations in the proposed epitope, MeV H and F (Edm and ICB323 strains) were co-expressed in Vero-hSLAM cells and fusion promotion was monitored 24h post-transfection in the presence or absence of the antibody. S3C and S3F Fig illustrate that mAb-1347 does not cross-react with MeV H, since cell-to-cell fusion remained essentially unaltered in the presence of the antibody. Since the putative mAb-1347 epitope in MeV H exhibited two substitutions compared to the sequence of CDV H, we generated a MeV H double mutant by changing these residues to their CDV counterparts. The MeV H-D128N/Y131F variant was bioactive when expressed with MeV F in receptor-positive cells but, strikingly, fusion promotion was completely inhibited by mAb-1347 (S3D and S3F Fig). When mapped in our previously generated 4HB-stalk structural model [31], the side chains of both residues are predicted to be solvent exposed, suggesting a direct contribution to the interaction with the mAb (S3E Fig).

The successful transfer of mAb-1347 membrane fusion inhibition to MeV H through epitope reconstruction implies that mechanistic insight into CDV fusion triggering can be extrapolated to related morbillivirus family members.

### mAb-1347 binding is not affected by the presence or absence of F

**Fig 4:**
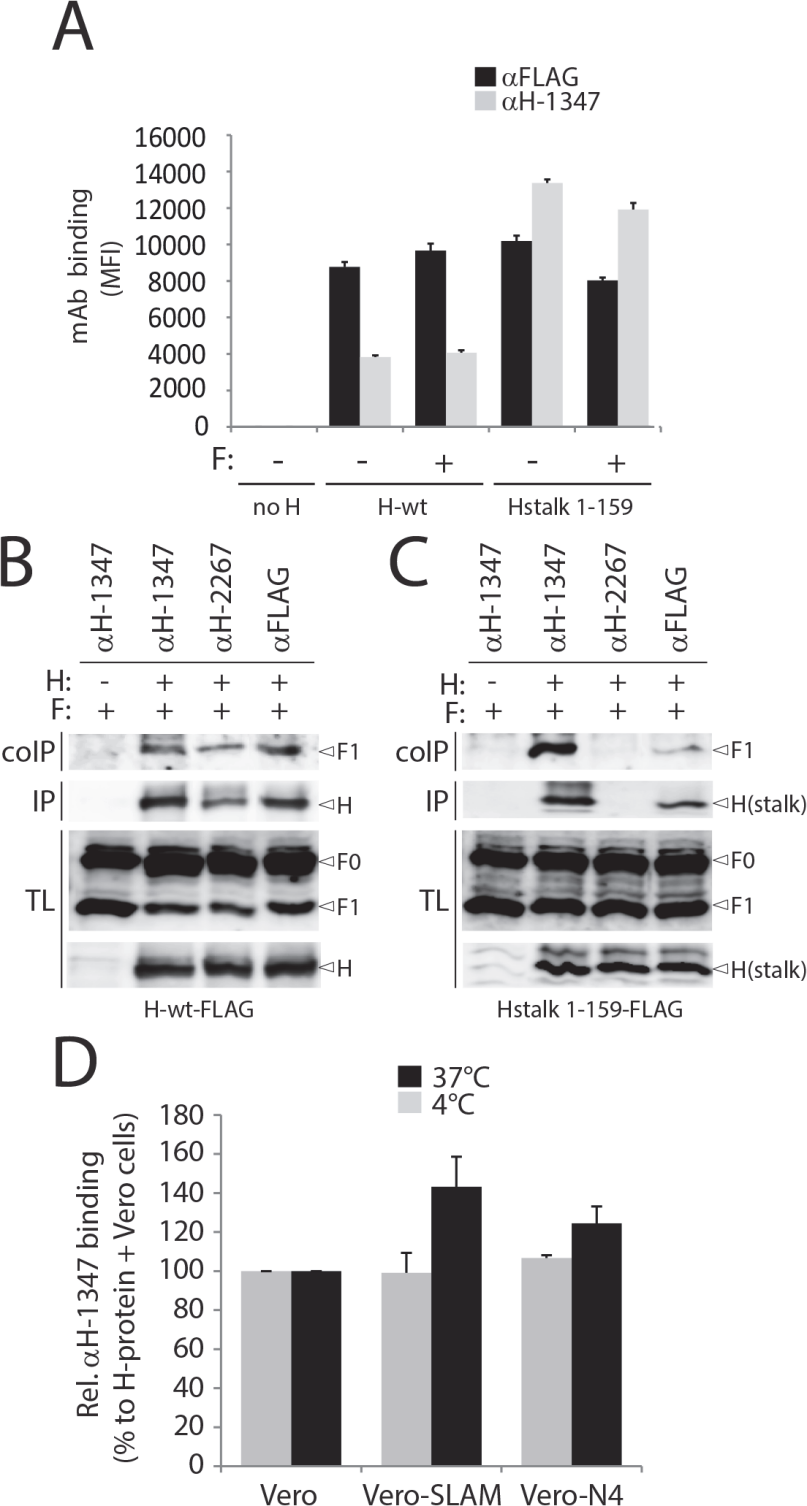
Receptor-induced conformational change in H detected by mAb αH-1347. (A) Reactivity to mAb αFLAG and αH-1347 of standard (H-wt) or headless (Hstalk) attachment proteins in the presence or absence of F-wt. After addition of the secondary antibody, MFI values were recorded by flow cytometry. H/F co-immunoprecipitation with different anti-CDV-H mAbs. (B) Cell surface assessment of H protein interaction with cleaved F-proteins. To stabilize H/F complexes, transfected Vero cells were treated with the membrane non-permeable cross-linker DTSSP and subsequently lysed with RIPA buffer. Complexes were then immunoprecipitated (IP) with anti-CDV-H mAb 3734 [61] and protein G-Sepharose beads treatment. Proteins were boiled and subjected to immuoblotting using a polyclonal anti-CDV-F antibody [71] to detect F antigenic materials (coIP). Co-IP F proteins were detected in comparison with F proteins present in cell lysates prior to IP by immuoblotting using the same anti-F antibody (TL, total lysate; F_0_, uncleaved F protein; F_1_, cleaved membrane-anchored F subunit). The specific mAb used for the immunoprecipitation step is indicated at the top of the gel. (C) Similar to (B) but with cell extracts obtained from Vero cells transfected with headless H and F. (D) Effect of receptor treatment on mAb αH-1347’s H-binding activity (at 4°C or 37°C). Vero cells expressing H-wt were co-cultured with Vero-SLAM, Vero-Nectin-4 or Vero cells together with mAb αH-1347. After addition of the secondary antibody, MFI values were recorded by flow cytometry. Means ± S.D. of data from three independent experiments performed in triplicates are shown.

The F-contact zone in MeV and CDV H supposedly includes residues encompassed in a linear section of the membrane-proximal stalk domain (110–118) [17, 31, 32, 57]. Our epitope mapping experiments place the mAb-1347 binding site (126–133) slightly membrane-distal from this candidate F-interaction domain. To investigate whether F-trimers sterically interfere with mAb-1347 binding activity, we performed IF and subsequently flow cytometry analyses on Vero cells expressing H-wt either alone or in combination with F. Results in Fig 4A document that regardless of the presence or absence of F, very similar MFI values were recorded. When we determined mAb reactivity to headless H (H-stalk 1–159) expressed alone or combined with F, identical MFI profiles were obtained (Fig 4A). Remarkably however, we noted a substantially higher mAb-1347 reactivity to headless CDV H than to full-length CDV H-proteins (Fig 4A), suggesting that the presence of the H-heads in a pre-receptor-bound conformation of the H tetramer may impair mAb-1347 binding.

To further test the notion that mAb-1347 can interact with H-tetramers even when complexed with F-trimers, we conducted H/F co-immunoprecipitation assays with mAb-1347 in receptor-negative Vero cells. Consistent with our cytometry results, F_1+2_ complexes were efficiently co-immunoprecipitated by mAb-1347 (recognizing the H-stalk) as well as the control 2267 and FLAG monoclonal antibodies (targeting the H-head) (Fig 4B). Co-immunoprecipitation experiments were repeated with the H-stalk 1–159 construct and results shown in Fig 4C indicate that F_1+2_ was again pulled down by both mAb-1347 and anti-FLAG. As anticipated, mAb-2267 could not co-immunoprecipitate F_1+2_ when co-expressed with headless CDV-H (Fig 4C).

Altogether, these data strongly support that mAb-1347 recognizes an F binding-competent H conformation that exists prior to receptor contact, but does not sterically interfere with the H/F hetero-oligomerization.

### Receptor engagement relocates CDV H-heads, exposing the C-terminal H-stalk section 126–133

Enhanced mAb-1347 reactivity to headless CDV-H constructs suggested that standard H-tetramers may assume a pre-receptor-bound conformational state in which at least one head domain partially shields the upper stalk region, blocking the epitope. Receptor binding may then induce conformational changes in the head domain that fully reveal the epitope.

To test this hypothesis, we expressed wild-type H-proteins in receptor-negative Vero cells. One day post-transfection, H-expressing cells were concomitantly treated with mAb-1347 and overlaid with Vero cells expressing a morbillivirus receptor (SLAM or Nectin-4), or standard receptor-negative Vero cells (1 hour at 4°C or 37°C). The reactivity of mAb-1347 with H-proteins was then monitored through immunostaining followed by flow cytometry. MFI values were first normalized for total cell surface expression (recorded by anti-FLAG mAb staining and flow cytometry) and then standardized to H-wt treated with regular Vero cells. Values remained mostly unaltered when cells were kept at 4°C (Fig 4D), but exposure to 37°C enhanced the mAb-1347 binding efficiency compared to values obtained for cells never exposed to receptor-positive cells (referred to as receptor-induced mAb-binding-enhancement (RBE) phenotype) (Fig 4D).

We previously reported that cell-cell fusion induction was dramatically enhanced when H/F-complexes were expressed in cells expressing SLAM compared to Nectin-4 [62]. Of note, we recorded a more pronounced RBE phenotype when H-tetramers were treated with SLAM than with Nectin-4, suggesting a direct correlation between fusion-support efficacy and the ability of H to exhibit the RBE phenotype. Consequently, our results support the hypothesis that mAb-1347 senses a significant conformational modification occurring in H upon receptor engagement.

Since H-tetramers are recognized to a certain extent by mAb-1347 prior to receptor binding (regardless of the presence of F) an H subpopulation may spontaneously assume a receptor-induced-like conformation. Alternatively, the pre-receptor-bound H conformation could mask some mAb-1347 epitope(s), allowing only partial recognition by the mAb. In either case, our data demonstrate that mAb-1347 efficiently detects a yet uncharacterized receptor-induced conformational change in H.

### CDV fusion activation requires at least two sequential conformational changes of H

**Fig 5:**
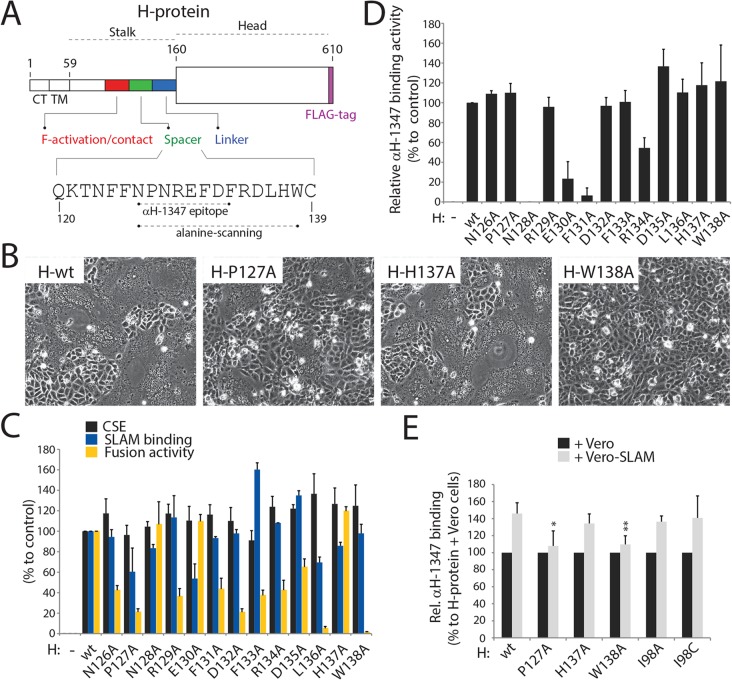
Most alanine H mutants spanning the “spacer” CDV H-stalk region are fusion-promotion impaired. (A) Schematic representation of the main functional domains of the full length CDV H-wt protein. The detailed primary sequence of the “spacer” section and the residues defining mAb αH-1347 epitope are shown. The H stalk-section scanned by alanine mutagenesis is also highlighted. (B) Syncytium formation assay. Cell-to-cell fusion activity in Vero-cSLAM cells triggered by co-expression of CDV H-wt or derived alanine variants with CDV F-wt. Representative fields of view of cell-cell fusion induced 24h post-transfection are shown. (C) Characterization of H mutants. Standard and derivative H mutants were expressed in Vero cells. One day post-transfection, cell surface expression (recorded by anti-FLAG staining followed by flow cytometry analyses), SLAM binding efficiency (calculated as described in the legend of Fig 1D, but without addition of mAbs before SLAM treatments) and fusion activity (monitored as described in the legend of Fig 1B) were determined. All values were normalized to H-wt. (D) Reactivity of standard and derivative H mutants to mAb αH-1347. MFI values were recorded by flow cytometry 24h post-transfection in Vero cells. (E) Effect of SLAM treatment on mAb αH-1347’s H-binding activity (37°C). The assay and values were determined as described in the legend of Fig 4D. Means ± S.D. of data from four independent experiments performed in triplicates are shown. To determine the statistical significance of differences between the standard and mutant H (127 and 138) data sets, unpaired two-tailed *t* tests were performed (*, P < 0.05; **P < 0.01).

We next asked whether the identified H-stalk microdomain may also impact the overall bioactivity of the H-tetramer. To address this question, 13 single alanine H-variants spanning residues 126–138 located in the upper “spacer” region of the H-stalk were generated (Fig 5A) and their ability to trigger F tested qualitatively and quantitatively in Vero-cSLAM cells. Although microscopically-detectable cell-to-cell fusion was observed with most H-mutants, quantitation revealed that only H mutants N128A, E130A and H137A induced fusion to levels comparable to those recorded for wild type H, whereas the remaining 10 mutants showed substantial functional deficiencies (Fig 5C).

Anti-FLAG-based monitoring of cell surface expression confirmed that all H-variants were properly surface-expressed (Fig 5C), suggesting that the loss of bioactivity was not due to gross protein misfolding. Assessment of SLAM binding revealed that most mutants likewise exhibited H-wt-like receptor binding. However, some showed slight (<50% of standard H) alterations, suggesting that parts of the 126–138 stalks section may affect the overall H conformation and influence receptor binding through long range effects (Fig 5C). Mutants H-N128A, E130A and F131A were substantially compromised in mAb-1347 binding, whereas all other mutants exhibited strong reactivity (Fig 5D). Combined with our previous results, these data support the conclusion that residues N128, E130, and F131 are directly recognized by mAb-1347.

We next tested the H alanine mutants for the mAb-1347 RBE phenotype. As before, H-variants were expressed in receptor-negative Vero cells and exposed to mAb-1347 and Vero cells expressing SLAM molecules or standard Vero cells at 37°C. For this experiment, we selected two hypo-fusogenic H-mutants (P127A and W138A) and one with wild-type-like fusion triggering activity (H137A) (Fig 5B and 5C), based on their ability to efficiently react with mAb-1347 (Fig 5D) despite the differences in F-triggering activity. Strikingly, both hypo-fusogenic variants exhibited a reduced RBE phenotype (Fig 5E), whereas the mutant associated with wt-like fusion-promotion (H-H137A) also displayed wt-like RBE (Fig 5E). Although the RBE-deficient phenotype of H mutant P127A may be partially attributed to slightly reduced SLAM binding activity, the H-W138A variant exhibited wt-like receptor binding (Fig 5C), suggesting an alternative mechanism blocking the RBE phenotype.

We have previously demonstrated that structural rearrangements within the central region of the H-stalks are strictly required for F-triggering. We therefore thought to determine whether the newly identified “head-stalk” conformational change occurred prior to, or after this “in-stalk” rearrangement. To address this question, we took advantage of H-mutants I98C and I98A, which are deficient in rearranging the central stalk section [17, 31, 33, 38, 64, 65], and assessed their ability to undergo receptor-induced “head-stalk” conformational changes. Strikingly, both mutants exhibited a wt-like RBE phenotype (Fig 5E), indicating that exposure of the 126–133 stalk section occurs prior to the structural rearrangements of the central region of the H-stalks.

Taken together, these data suggest that (i) the 126–138 section contributes to the folding of H-tetramers into fusion triggering-competent states, (ii) receptor-induced conformational changes exposing the H 126–138 upper stalk section are required for productive F-triggering, and (iii) the “head-stalk” rearrangements precede the “in-stalk” conformational changes.

### Stalk-elongated CDV H-proteins require the “head-stalk” conformational change to activate F

Whereas MeV H-variants lacking the head domains retained receptor-independent F-triggering bioactivity, stalk-elongated (heads-carrying) versions did not [47, 57]. Rather, these stem-elongated H-constructs still required the presence of receptor to trigger F [57]. Since insertions in MeV H were added N-terminal to the spacer region (between residues 118 and 119), we speculated that the bioactivity of such H-elongated mutants remained dependent on the presence of the receptor because the extended stalks remain able to assume proper pre-F-triggering folds and therefore still rely on the coordinated “head-stalk” conformational changes to activate F.

**Fig 6:**
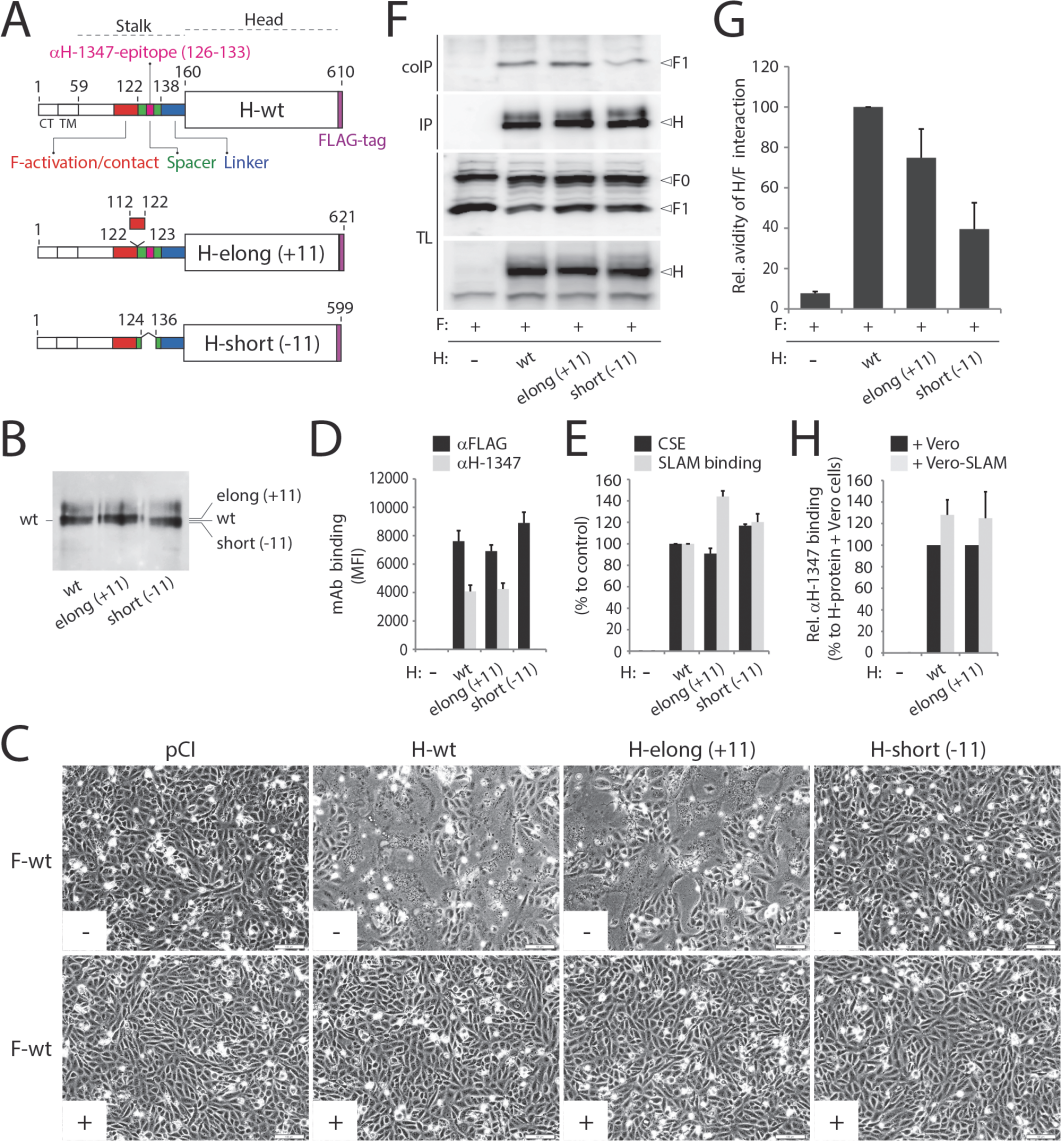
Functional and biochemical characterization of stalk-elongated and shortened engineered H variants. (A) Upper part: schematic representation of the main functional domains of the full length CDV H-wt protein. Middle part: schematic representation of the H-elongated construct with the precise position of the insertion indicated. The 11 residues inserted are derived from the N-terminal stalk “contact” section 112–122 that putatively assume a helical fold with an 11-mer repeat. Lower part: schematic representation of the H-shortened version with the precise position of the deletion indicated. (B) Immunoblotting of standard and size-modulated H proteins. Antigenic materials ran in an 8% SDS-Page gel under reducing conditions were detected using a polyclonal anti-H antibody. (C) Syncytium formation assay. Fusion activity in Vero-cSLAM cells triggered by co-expression of CDV H (or size-modulated mutants) and CDV F in the presence (+) of absence (-) of mAb αH-1347. Representative fields of view of cell-cell fusion induced 24h post-transfection are shown. (D) Reactivity of H-wt and size-modulated H variants to mAbs αFLAG and αH-1347. Vero cells were transfected with the different H plasmids and, after addition of the secondary antibody, MFI values were recorded 24h post-transfection. (E) SLAM-binding activity of H mutants. Standard and H mutants were expressed in Vero cells. One day post-transfection, cell surface expression (CSE; recorded by anti-FLAG staining followed by flow cytometry analyses) and SLAM binding efficiency (calculated as described in the legend of Fig 1D, but without addition of mAbs before SLAM treatments) were determined. All values were normalized to H-wt. (F) Co-IPs, performed as described in the legend of Fig 4B, were obtained 24 hours post-transfection of cell extracts of Vero cells transfected with standard H or derivative mutants and F-wt-expressing vectors. (G) Semiquantitative assessment of F/H avidity of interactions. To quantify the avidities of F_1_-H interactions, the signals in each F_1_ and H bands were quantified using the AIDA software package. The avidity of F_1_-H interactions is represented by the ratio of the amount of coimmunoprecipitated (coIP) F_1_ over the product of F_1_ in the cell lysates divided by the ratio of the amount of immunoprecipitated H over the product of H in the cell lysate ((coIP F_1_/TL F_1_)/(IP H/TL H)). Subsequently, all ratios were normalized to the ratio of the wild-type F-H interactions set to 100%. Averages represent at least two independent experiments. (H) Effect of SLAM treatment on mAb αH-1347’s H-binding activity. The assay and values were determined as described in the legend of Fig 4D (37°C). Means ± S.D. of data from two independent experiments performed in triplicates are shown.

To test this hypothesis, we inserted a segment of 11 residues (putatively representing 1 complete turn of the helical wheel of this stalk portion) into the CDV H-stalk between positions 122 and 123 (H-elong (+11)). We also constructed a second mutant in which stalk residues 125–135 are deleted, thus removing the mAb-1347 epitope from H (H-short (-11)) (Fig 6A). Slight mobility shifts were found when fractionating both engineered H proteins in SDS-PAGE followed by immunoblotting (Fig 6B). The results summarized in Fig 6 indicate that H-short (-11) completely lacked bioactivity regardless of the presence or absence of the receptor (Fig 6C), despite efficient cell surface expression and SLAM binding activity (Fig 6D and 6E). In contrast, the elongated H version, which likewise remained intracellular transport-competent and bound SLAM and mAb-1347 efficiently (Fig 6D and 6E), promoted membrane fusion in the presence of SLAM (Fig 6C). However, bioactivity was entirely inhibited by mAb-1347 (Fig 6C). Co-immunoprecipitation experiments revealed that H-elong (+11) exhibited proper F-binding activity, whereas H/F interactions were impaired when F was co-expressed with the shortened version of H (Fig 6F and 6G), as previously demonstrated for an equivalently shortened MeV H mutant [56]. Importantly, H-elong (+11) displayed wt-like mAb-1347 RBE (Fig 6H).

Overall, the fact that H-short (-11) remained transport competent but fusion promotion-inactive confirms the essential role of the stalk region in ensuring sterical compatibility of morbillivirus H and F. Efficient RBE recorded with H-elong (+11) confirmed that the stalk-elongated construct can proceed to productive F-triggering while fully relying on the “head-stalk” conformational change to trigger F refolding.

### mAb-1347 to H stoichiometry required for fusion inhibition

The above results suggest that at least one mAb-1347 epitope must remain inaccessible to mAb binding in the pre-receptor-bound H conformation. This inferred that a “less-than-parity” stoichiometry of mAb to H-monomer may be sufficient to inhibit fusion. On the other hand, we cannot exclude the possibility that receptor binding-dependent unmasking of mAb-1347 epitope(s) opens a window of opportunity for additional mAb-1347 interaction (leading to a critical 1:1 stoichiometry).

**Fig 7:**
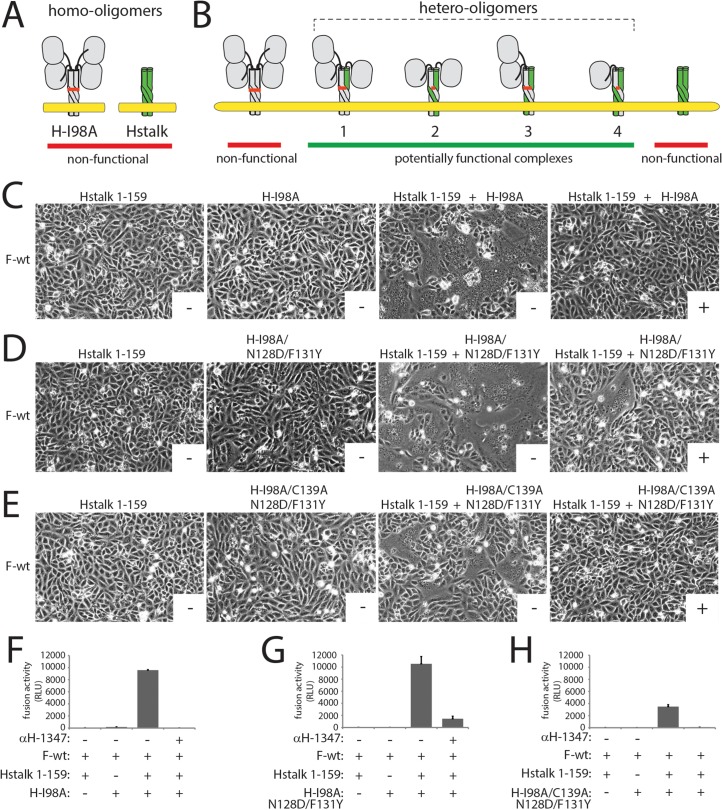
Investigation of the mAb αH-1347 to H stoichiometry required for membrane fusion inhibition. (A) Cartoon representation of the CDV H-wt tetramer and derived “headless” variant in a putative pre-F-triggering state. The reported I98A mutation shown to abrogate F-triggering without impairing H/F interaction is also shown in the full length H-wt protein [17, 64]. (B) Representation of the different hetero-oligomeric assemblies that may emerge from H-98A and H-stalk co-expressing cells. (C-E) Syncytium formation assay. Fusion activity in Vero-cSLAM cells triggered by co-expression of CDV H-98A and F-wt, or headless H and F-wt, or H-I98A and headless H and F in the presence (+) of absence (-) of mAb αH-1347. Representative fields of view of cell-cell fusion induced 24h post-transfection are shown. (F-H) Quantitative fusion assay. The fusion promotion efficiency of each H/F combinations was determined as described in the legend of Fig 1B. Means ± S.D. of data from three independent experiments performed in triplicates are shown.

To shed light on mAb-1347 to H stoichiometry requirements for fusion-inhibition, we adapted to the CDV system a previously reported H trans-complementation assay first developed for MeV ([38] and Fig 7A and 7B). We based the assay on two reportedly inactive H-mutants: a FLAG-tagged headless CDV-H (unable to activate the highly stable F-wt) and the HA-tagged H-I98A variant (F-trigger-defective, [17, 64]). While cell-to-cell fusion remained absent when Vero-SLAM cells expressed F-wt together with either one of the H-mutants, fusion was fully restored when cells co-expressed F and both H-mutants (Fig 7C and 7F). Cell surface co-immunoprecipitation experiments confirmed that mixed oligomers readily formed (S4A Fig). As expected, restored fusion activity was efficiently blocked by mAb-1347 (Fig 7C and 7F). These results indicate that headless and I98A H-mutants efficiently trans-complemented their fusion-deficiencies when assembled into mixed oligomers. In addition, these data confirmed the earlier finding that receptor binding to less than four H-heads per tetramer is sufficient to initiate F-refolding [38, 66].

We next introduced the N128D and F131Y dual substitutions into the H-I98A background and repeated the trans-complementation assay. In H-wt, these substitutions ablated mAb-1347 binding (S4B and S4C Fig). As anticipated from our earlier finding that mutations D128N and Y131F do not impair H bioactivity (S3C Fig), co-expression of headless H with H-I98A/N128D/F131Y resulted in restoration of membrane fusion activity through trans-complementation (Fig 7D and 7G). Remarkably, fusion activity promoted by functional hetero-oligomeric H-proteins was drastically reduced in the presence of the mAbs (Fig 7D and 7G). As noted previously for MeV H hetero-oligomers, complementation in this setting could be based on mixing of H monomers (resulting in hetero-dimers) or dimers (resulting in homo-dimers/hetero-tetramers). To distinguish between these alternatives, we added a supplementary C139A substitution to the H-I98A/N128D/F131Y triple mutant, following the previously established disulfide bond engineering approach to selectively test trans-complementation on the homo-dimer/hetero-tetramer level [38]. This strategy reportedly generates a population of hetero-oligomers with only two available mAb-1347 epitopes, both located in the H stalk 1–159 monomers. Although trans-complementation efficiency under these conditions was reduced, mAb-1347 nevertheless completely blocked membrane fusion (Fig 7E and 7H).

Together with the result that mAb-1347 epitopes are only partially accessible in a pre-receptor-bound H conformation (based on RBE and coIP assays) but sufficient for blocking viral cell entry (based on neutralization assays), these findings suggest that (i) a “less-than-parity” mAb-to-H monomer stoichiometry leads to fusion inhibition, and (ii) the H-heads in a putative “heads down” conformation mask some mAb-1347 epitopes.

## Discussion

In the present study, we report the characterization of a monoclonal antibody (mAb-1347), which targets the C-terminal “spacer” module (aa 126–133) of the CDV H-stalk domain. Binding of this mAb potently inhibited bioactivity of both standard and “headless” CDV H-variants without interfering with either receptor-binding or F-interaction abilities. Remarkably, our data demonstrated that mAb-1347 exhibited an enhanced reactivity not only with head-truncated H-constructs, but importantly also with standard and stalk-elongated CDV H-tetramers after exposure to receptors (RBE phenotype). Although we cannot entirely exclude the possibility that a conformational change in the stalk is triggered by the mAb itself, the fact that enhanced antibody binding activity coincides specifically with receptor binding argues against this hypothesis. Our data therefore spotlight an as yet unrecognized receptor-induced structural rearrangement of the morbillivirus H-protein. Since previously reported “open stalk”-defective H-mutants likewise displayed the RBE phenotype, our findings furthermore reveal the sequence of H conformational changes that are required to translate receptor binding into F-activation.

**Fig 8:**
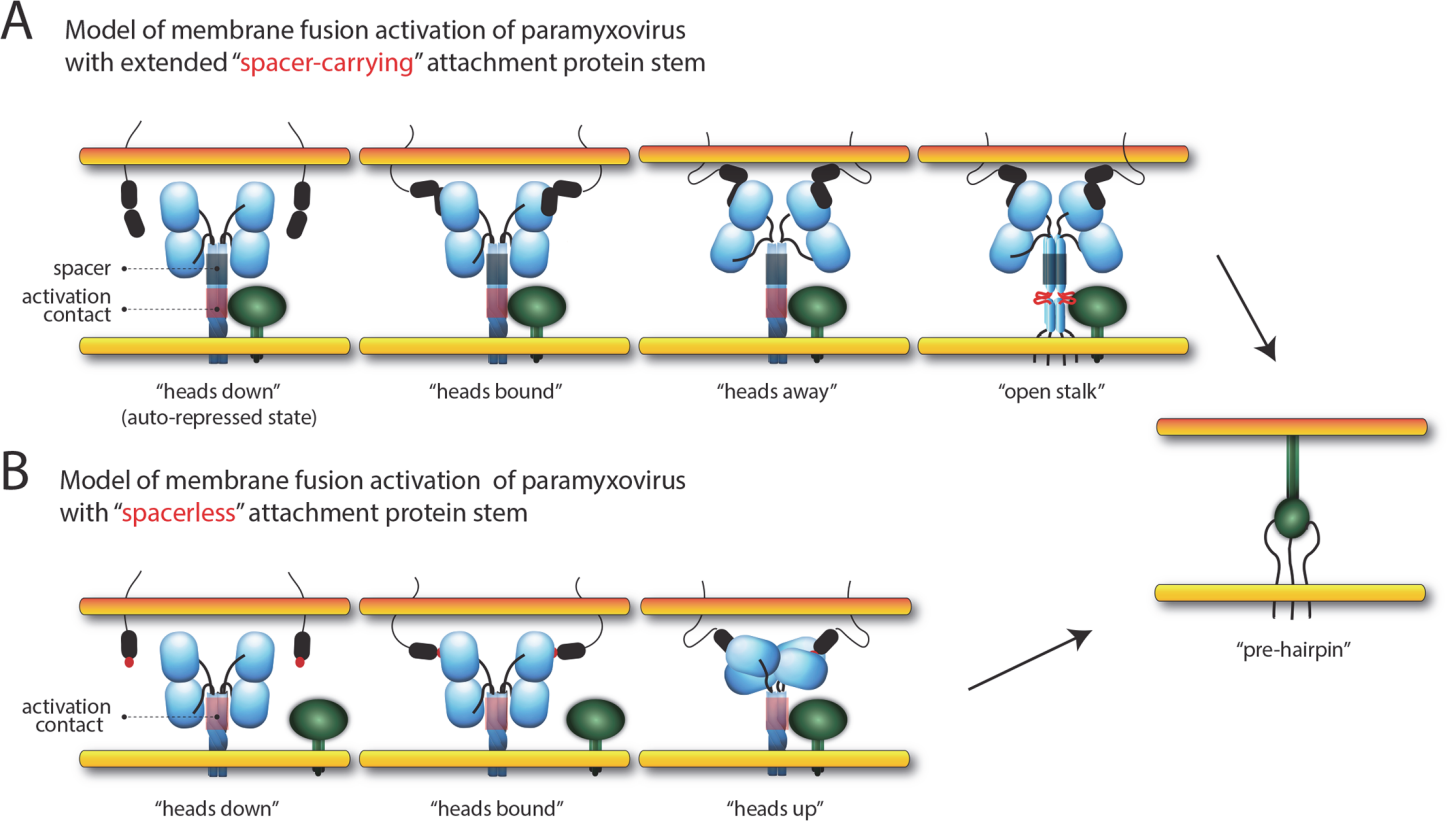
Models of paramyxovirus membrane fusion activation. (A) Summary of the putative mechanism of membrane fusion activation for morbilliviruses (carrying the “spacer” module in the H-stalk). In the first panel, the cartoon represents one H-wt tetramer (in blue) assuming the auto-repressed state not yet bound to its cognate receptor (*i*.*e*. SLAM; represented by black ovals anchored in the target plasma membrane). The associated trimeric fusion protein (F) in the pre-fusion conformational state is represented in green. Because the “4-heads-down” configuration determined for NDV HN [36] contains elements that fit with the proposed pre-receptor-bound locked H-structure, we arbitrarily illustrated H in this conformation. In this state, the two “lower” H-heads are partially covering the “spacer” stalk microdomain (green box) where the mAb αH-1347 epitopes also locate. These “head-to-spacer” contacts are critical in stabilizing the auto-repressed H state. Because the H-stalk “spacer” module maps membrane-distal from the candidate F-binding/activation regulatory segment (red box), H/F assembly can occur even prior to receptor binding. In the second panel, both accessible “upper” heads engage with the receptor. Conversely, distance and/or physical constraints may prevent efficient receptor binding to the “lower” head units. In the third panel, because both monomeric head units within each dimers may assemble into structurally stable complexes (or may require some adjustment prior to achieve stable dimeric units [75]), the contact of the “upper” heads with the receptor leads to a rearrangement of the dimers that automatically relocates the “lower” heads “away” from the stalk (achieving a putative “heads away” structural intermediate). This step thus disrupts the critical “head-to-spacer” contacts and leads to the “de-activation” of the auto-repressed state. In the fourth panel, the stalk is now free to refold into the F-triggering competent state. The latter includes “structural flexibility” or “opening” of the central section. In the fifth panel, upon H-mediated activation, F achieves the pre-hairpin intermediate structural state bridging the viral envelope and the target cell plasma membranes (color-coded in yellow and orange, respectively). Basics of this model was recently hypothesizes for CDV [68] and MeV [48] (referred to as “safety catch” in the latter study). (B) Summary of the putative mechanism of membrane fusion activation of PIV5 and NDV (expressing attachment protein with short “spacerless” stalks). In the first panel, the pre-receptor-bound state of HN is represented in the “4-heads-down” configuration not yet bound to its cognate receptor (*i*.*e*. sialic acid; represented by red spheres attached to membrane bound molecules). As a major difference with morbillivirus attachment proteins, HN-stalks do not carry the analogous “spacer” segment that locate membrane-distal to the F-binding/activation sites (red box). Consequently, the two backfolding dimeric head units directly cover the F-binding/activation sites, which prevent HN/F assembly prior to receptor engagement. In the second panel, both accessible “upper” heads engage with the receptor. In the third panel, the contact of the HN-Heads with the receptor triggers a large-scale conformational change that switches the heads from the “down” to the “up” configuration. The latter structural state implies a tetrameric assembly of the heads positioned above the stalk. Consequently, the F-binding/activation sites are unmasked, which in turn allow for HN/F interactions and subsequent “induced fit” mechanism that ultimately lead to F-triggering. In the fourth panel, F achieves the pre-hairpin intermediate structural state bridging the viral envelope and the target cell plasma membranes (color-coded in yellow and orange, respectively). This model is referred to as the “stalk-exposure/induce fit” model [40, 46].

While intracellular assembly of morbillivirus H//F complexes [48, 67] directly challenged the “stalk exposure/induced fit” hypotheses of paramyxovirus cell entry [40, 46], the recently proposed model of membrane fusion activation mediated by CDV [68] and MeV [48] (referred to as the “safety catch” model in the latter study) reconciled the different datasets. In the present study, we not only deliver tangible mechanistic insight in support of the safety catch hypothesis, but also substantially extend the model by unravelling the molecular nature governing the dynamics of morbillivirus membrane fusion triggering; prior to receptor-binding, H-tetramers initially fold into an auto-repressed conformational state, where the inherent F-triggering activity of the stalk is silenced by a specific positioning of the head domains. Importantly, we propose that this H auto-repressed state does not necessarily require covering of the F-binding/activation sites in order to prevent untimely H/F interaction and subsequent premature F-activation. Rather, receptor-contact at the cell surface of intracellularly preformed H/F complexes leads to re-positioning of the head domains (referred to as the “head-stalk” conformational change), which consequently releases the conformational lock on the stalk domain. As a result, the stalk is freed to spontaneously undergo the change to the “open stalk” conformation, which is required to trigger the initiation of the F-refolding cascade (Fig 8A).

It is tempting to speculate that the proposed auto-repressed state of H resembles the “heads down” configuration obtained for PIV5 and NDV HNs [34, 36]. Indeed, the physical contacts of the “lower” heads with the membrane-distal stalk region may be key to stabilize the auto-repressed conformational state. In contrast to morbillivirus H-proteins, however, HN-stalks do not display an analogous stalk spacer section located C-terminal to the 4HB portion, but short helices and/or flexible linkers that likely directly connect the globular head domains to the 4HB section [34]. Therefore, variation of the attachment protein stalk length among different members of the paramyxovirus family may govern discrete modes of envelope glycoprotein interactions and mechanisms of membrane fusion activation, despite a very similar overall structural organization. Indeed, attachment proteins characterized by extended (“spacer-carrying”) stalks can accommodate intracellular H/F association and thus may depend on the “safety catch” model for F-activation (Fig 8A). Conversely, attachment proteins with a short (“spacerless”) stem feature head domains directly covering the F-binding/activation sites, which consequently prevent intracellular F interaction. These attachment proteins may therefore rely on “stalk-exposure” and subsequent “induced fit” models to trigger F [40] (Fig 8B). Viral escape mutants to a fusion-inhibitory synthetic antibody targeting the PIV5 HN-stalk, which presumably blocks interaction with F, consistently exhibited mutations in the candidate F-binding/activation sites, thereby confirming this section as a key functional microdomain in spacerless HN-stalks [69].

The engineered spacerless morbillivirus H-variant and alanine H-mutants spanning the spacer domain were impaired in fusion-promotion. Strikingly, the spacer alanine mutants additionally exhibited a deficiency to properly undergo the RBE phenotype. These data thus spotlight a critical dual function of the morbillivirus H-stalk spacer microdomain: (i) residues within this section contribute to the folding of H-tetramers into the correct pre-receptor-bound conformation and (ii) they govern proper initiation of the newly identified head-stalk conformational change. These findings thus favor the hypothesis of a critical head-to-spacer interaction, which is instrumental to stabilize the auto-repressed state. Mutations in spacer may modulate the avidity of interactions with the contacting head(s), consequently steadily locking or de-activating the stabilized state. In either case, membrane fusion activation will be inhibited, since H-proteins are either unable to refold or will experience the second “open stalk” conformational change prematurely. Collectively, the C-terminal H-stalk spacer microdomain emerges as an essential molecular determinant controlling the activation and de-activation stages of the auto-silenced pre-triggering conformation of H.

It was demonstrated that elongated MeV H-constructs with segments inserted C-terminal to stalk residue 118 remain bioactive [57]. Although their membrane-proximal stem regions (aa 59–118) are constitutively exposed, such mutants still required receptor binding to activate the fusion process. Likewise, the stalk-elongated CDV H-variant generated in this study remained fully bioactive and experienced the receptor-induced head-stalk conformational change to trigger F. In both the MeV and CDV elongated H-constructs, the insertions were located N-terminal to the spacer region. Since both the heads and the functional spacer microdomain were thus moved by the insertion as a single module, we hypothesize that stalk-elongated H-tetramers still efficiently assume the auto-repressed state, which further strengthens the hypothesis of the head-to-spacer interaction exerting an essential role in the spatiotemporal regulation of the membrane fusion process.

Liu and colleagues recently proposed a three-step receptor-induced mechanism of NiV G-mediated membrane fusion activation, which interestingly also included a final head-stalk-like conformational change as a critical step leading to F-triggering [49]. Although these data suggest a common mechanism used by henipaviruses and morbilliviruses for cell entry, the authors identified the presumptive head-stalk-like conformational change using a polyclonal antibody raised against an engineered full-length G-stem construct. Hence, in contrast to our work, the microdomain of the G-stalk that might be exposed upon receptor binding remained undetermined, which complicated the interpretation of the model. Different from the morbillivirus safety catch model, the authors suggested a bi-dentate attachment protein/F interaction mechanism to exclude premature F-activation upon intracellular G/F interaction: F-trimers may initially bind to the attachment protein head domains and switch to interaction with the stalks upon receptor binding [49]. While this could be true for the henipaviruses, data obtained for CDV and MeV H-proteins rule out this hypothesis for the morbillivirus fusion complexes. Firstly, H-variants with an elongated stalk (and demonstrated head-lift) remained capable of F binding [57]. Secondly, the H-stalk mutant F111A lost binding competence to proteolytically matured F-trimers even in receptor-negative cells [57]. Thirdly, head-truncated H-constructs retained intracellular F interaction [48]. While these findings collectively support a single binding interface governing morbillivirus envelope glycoprotein interactions, additional experiments are needed to determine whether NiV glycoproteins either rely on a bi-dentate mechanism for cell entry, or more closely mimic the morbillivirus entry machinery through intracellular prefusion F binding directly to the G-stalk, which is auto-silenced until receptor contact.

Based on EM data, Giu and colleagues recently proposed that HPIV3 HN/F complexes can form prior to receptor engagement and that the heads up configuration itself is insufficient to activate F [70]. Upon receptor contact, the HN-heads may transmit a signal for F-activation, possibly through an oligomerization mechanism. While possible, this hypothesis is largely supported by data from the same group suggesting clustering of preformed HN/F complexes upon receptor engagement [58, 59]. At present, however, direct experimental support is lacking to confirm that monomeric, dimeric or tetrameric HN-structures indeed switch to higher order oligomers in a receptor binding-dependent manner to achieve complexes productive for F-triggering.

In conclusion, our data highlight two sequential conformational changes in morbillivirus H-tetramers that together constitute the mechanistic core of the molecular link between receptor binding and F-triggering. Additionally, our findings provide first molecular evidence that both of these rearrangements are triggered upon receptor-induced de-activation of an auto-repressed conformational state of the H-stalk assumed prior to receptor binding. This locked state likely originates from a critical head-to-spacer interaction that allows intracellular H/F assembly, but prevents premature F-activation. Lastly, beyond having identified the spacer module as the key element in the H-stalk coordinating the dynamics of the safety catch mechanism, our data further reveal that despite possible strong overall structural conservation between different paramyxovirus envelope glycoproteins, the presence or absence of the spacer domain in the attachment protein stalk emerges as a key indicator for the F-triggering strategy applied by individual paramyxovirus family members.

## Materials and Methods

### Cell cultures, transfections and viruses

293T cells (ATCC), Vero (ATCC CCL-81) and derivative Vero cells expressing the canine SLAM (Vero-cSLAM, kindly provided by Yusuke Yanagi, Kyushu University, Japan) or the Nectin-4 (Vero-Nectin-4, [62]) receptors were grown in Dulbecco's modified Eagle's medium (Gibco, Invitrogen) with 10% fetal calf serum at 37°C in the presence of 5% CO_2_. Cells were transfected using TransIT-LT1 (Mirus). The MVA-T7 recombinant vaccinia virus was used for a quantitative cell-cell fusion assay and was kindly provided by B. Moss, NIH, Bethesda, MD. The recombinant A75/17-CDV, containing an additional RFP gene (recA75/17^red^), was amplified and titrated in Vero-cSLAM cells.

### Construction of expression plasmids

All single (and multiple) substitutions performed in pCI-CDV-H and pCI-CDV-F (derived from hemagglutinin of the A75/17 CDV strain [71]) were obtained using the Quick Change lightning site-directed mutagenesis kit (Stratagene). Mutations were also performed in measles virus attachment protein-expression vectors (pCI-MeV-H derived from the ICB323 and Edmonston strains (kindly provided by Jürgen Schneider-Schaulies, Würzburg, Germany)). The pCI-H301F vector was previously described and encodes the hemagglutinin protein derived from a fox-isolated CDV strain (W10/301/red-fox/Ch/2010-JF810106 and referred in this study to as 301F) [72]. To produce a soluble form of H, the complete ectodomain was cloned in-frame with the IgK signal peptide. In addition, to maximize the prospect of preserving the H-tetramer oligomeric state in its soluble form, the GCN4 peptide was fused N-terminally as well as a hexahistidine tag (6xHis) [31]. FLAG-tag insertions (DYKDDDK) at the C-terminal region of the CDV H-proteins were performed by site directed mutagenesis, using the same kit as described above. All primers are available upon request.

### Transfections and luciferase reporter gene content mix assay

Vero cells, in 6-well plates at 90% confluency, were co-transfected with 2 μg of different pCI-F constructs, 1 μg of the various pCI-H plasmids with 9 μl of TransIT-LT1 (Mirus). Vero cells in 24 wells were transfected with 1μg of the various pCI-H plasmids with 3 μl of TransIT-LT1 (Mirus). All transfections were performed according to the manufacturer’s protocol. In some experiments, phase contrast pictures were taken 24 h post-transfection with a confocal microscope (Olympus, Fluoroview, FV1000).

The quantitative fusion assay was performed as described previously [73, 74] with subtle modification. Briefly, Vero cells were co-transfected with the F and H expression plasmids and 0.1 μg of pTM-Luc (kindly provided by Laurent Roux, University of Geneva). In parallel, separate 6-well plates of Vero-cSLAM cells at 30% confluency were infected with MVA-T7 at a multiplicity of infection (MOI) of 1. After overnight incubation, both cell populations were mixed and incubated at 37°C. 2.5 hours later, the cells were lysed using Bright Glo Lysis Buffer (Promega), and the luciferase activity was determined using a luminescence counter (PerkinElmer Life Sciences) and the Britelite reporter gene assay system (PerkinElmer Life Sciences).

### Soluble H-mutants production and immunoprecipitation assay

293T cells were transfected with 10μg of various expression vectors. 72h post-transfection, the supernatant was harvested and concentrated using 10 kDa cutoff filtration columns (Millipore). Subsequently, equal aliquots of supernatants were immunoprecipitated for 2 hours with either mAb 1347, 1C42 or 2267 (1:1000) [61] or anti-histidine mAbs (AbD Serotec) (1:500). This was followed by adding protein G-Sepharose beads overnight (GE-Healthcare) and subsequently fractionated in 10% NuPAGE Bis-Tris Mini gels (life technologies) under regular reducing conditions. Immunoprecipitated H-proteins were finally revealed by western blotting, as described, using a polyclonal anti-H antibody or anti-GCN4 antibody (Santa Cruz Biotechnology) (1:1000).

### Western blotting

Western blots were performed as previously described [29]. After overnight incubation on a rotor at 4°C, the lysates were cleared by centrifugation for 3min at 4°C, and washed three times with RIPA (10mM Tris, pH7.4, 150mM NaCl, 1%deoxycholoate, 1% Triton X-100, 0.1% sodium dodecyl sulfate (SDS)) containing protease inhibitor (Roche, complete mix). The supernatant was mixed with an equal amount of 2x Laemmli sample buffer (Bio-Rad) containing 5% β-mercaptoethanol, subsequently boiled at 90°C for 5 min and fractionated on NuPAGE Bis-Tris Mini gels (life technologies) under regular reducing conditions. Separated proteins were transferred to nitrocellulose membranes by electroblotting. The membranes were then incubated with the polyclonal anti-CDV-H, anti-CDV-F [71] or anti-GCN4 antibody (Santa Cruz Biotechnology) (1:1000). Following incubation with a peroxidase-conjugated secondary antibody, the membranes were subjected to enhanced chemiluminescence (ECL) kit (Amersham Pharmacia Biotech) according to the manufacturer's instructions.

### F/H co-immunoprecipitation assay

CDV F/H co-immunoprecipitations, initially developed by Paal and colleagues [57], were performed as previously described [28] with the following modifications. Vero cells in a 6-well plate format were transfected in duplicate with 2μg of CDV H-protein expressing plasmids (or derived variants) and 2μg of CDV F-protein expressing plasmids. 24h post-transfection, the cells were washed with cold PBS and treated with DTSSP (1mM final concentration in PBS, ProteoChem). Cells were subsequently lysed in RIPA buffer (10mM Tris, pH 7.4, 150mM NaCl, 1% deoxycholate, 1% Triton X-100, 0.1% sodium dodecyl sulfate (SDS)) containing protease inhibitor (Roche, complete mix). Cleared lysates (20,000 X g; 20 min, 4°C) were incubated 2–4 hours with the indicated monoclonal antibody (1:500), followed by overnight incubation with immunoglobulin G-coupled Sepharose beads (GE-Healthcare). The samples were then subjected to Western blot analysis as described above using either a polyclonal anti-HA antibody (Covance), a polyclonal rabbit anti-F antibody or a polyclonal anti-H antibody.

### Immunofluorescence staining and flow cytometry

Vero cells were transfected with 1μg of various H-expressing proteins alone or combined with 1μg F-expressing DNA plasmids. One day post transfection, unfixed and unpermeabilized cells were washed twice with cold phosphate buffered saline (PBS) and subsequently stained with the various antibodies (1:1000) for 1h at 4°C. The anti-CDV F mAbs 4941 and 3633, anti-CDV H mAb 1347, anti-FLAG mAb (Sigma Aldrich) or anti-HA mAb (Covance) (1:1000) were employed. This was followed by washes with cold PBS and incubation of the cells with Alexa-fluor 488-conjugated secondary antibody (1:500) for 1h at 4°C. Cells were subsequently washed 2 times with cold PBS and consequently detached from the wells by adding PBS-EDTA (50μM) 20min at 37°C. The mean fluorescence intensity (MFI) of 10’000 cells was then measured by using a BD LSRII flow cytometer (Becton Dickinson).

### SLAM binding activity

10μg of a soluble HA-tagged SLAM protein-expressing plasmid (of canine origin) was transfected in 293T cells seeded in petri dishes. Four days later, 10ml of the supernatant was harvest and concentrated using 10 kDa cutoff filtration columns (Millipore). Then, H-expressing receptor-negative Vero cells were incubated 1h at 4°C with concentrated soluble SLAM molecules (at a dilution of 1:20), followed by two PBS washes and treatment with monoclonal anti-HA (1:1000) (Covance) 1h at 4°C. Cells were then incubated with Alexa-fluor 488 conjugated secondary antibody (1:500) (Invitrogen) 1h at 4°C and were submitted to flow cytometry as described above. Relative SLAM-binding activity of the H-proteins was determined by MFI values recorded with the anti-HA antibody (SLAM-binding efficiency) that were normalized to MFI values obtained with anti-FLAG antibody (cell surface expression) of each H variants. Values were finally normalized to the H-wt/SLAM binding efficiency (set at 100%).

### Virus neutralization assay

One hundred infectious units of recA75/17^red^ (titer 4,7x10^6^ IU/μl) were incubated with the indicated dilution of the neutralizing mAb-1347 for 1h on ice. The virus-antibody mixture was then added to Vero cells and plates were incubated at 37°C. Four hours post-infection, medium was replaced by DMEM containing 1% agar. The numbers of syncytia were counted under a fluorescence microscope 72 hours of post-infection. Values were then normalized to recA75/17^red^-induced number of syncytia obtained in the absence of any mAb treatments (set at 100%).

## Supporting Information

S1 Fig Characterization of mAb αH-1347. (A) The sol H-ecto construct was expressed three days in 293T cells. Antigenic materials from harvested supernatants were run in an 8% SDS-Page gel under reducing conditions and subsequently detected using different monoclonal antibodies (as indicated in the text). (B) Schematic representation of the full length CDV H protein with the main functional domains in the stalk region color-coded. The length and the functional domain(s) deleted of each headless H variants are represented below. (C) The wt H protein and respective heads-deleted versions were expressed one day in Vero cells. Reactivity of standard and derivate headless H mutants to mAb αH-1347 and αFLAG were then determined by flow cytometry analyses after addition of the secondary antibody. Means ± S.D. of data from three independent experiments performed in triplicates are shown.
(TIF)

S2 Fig Biochemical characterization of Hstalk 1–159. (A) The H construct was expressed one day in Vero cells and its oligomeric state subsequently investigated by immunoblotting using an anti-H polyclonal antibody of gels ran under (A) denaturating and reducing conditions, (B) denaturating and non-reducing conditions and (C) native conditions. The molecular weight of the markers is shown on the left side of each gel. H1: monomeric H; H2: dimeric H; H4: tetrameric H.
(TIF)

S3 Fig Epitope αH-1347 reconstruction in MeV H leads to complete inhibition of membrane fusion by mAb αH-1347. (A) Sequence alignment of the mAb αH-1347 epitope of various members of the morbillivirus genus. (B, C and D) Syncytium formation assay. Vero-cSLAM cells were transfected with the indicated H-expressing plasmids together with the indicated F-expressing plasmid in the presence or absence of mAb αH-1347. Representative fields of view of cell-cell fusion induced 24h post-transfection are shown. (E) Homology structural model of the 4-helical bundle CDV H-stalk region [31]. The atomic coordinates of the visible residues involved in mAb αH-1347 epitope are color-coded in red. In addition, the two residues that were shown to strongly influence mAb αH-1347 binding activity are highlighted in green. (F) Quantitative fusion assay. The fusion promotion efficiency of each H/F combinations (in the presence or absence of the mAb) was determined as described in the legend of Fig 1B. Means ± S.D. of data from three independent experiments performed in triplicates are shown.
(TIF)

S4 Fig Efficient hetero-oligomeric assembly between headless and standard or H variants. (A) Cell surface co-immunoprecipitation experiments were performed to assess the formation of H hetero-oligomeric complexes. HA or FLAG-tagged H variants were expressed alone, or in combination, in Vero cells (as indicated in the upper panel). One day post-transfection, Vero cells were decorated with anti-FLAG mAb for 1 hour at 4°C. Cells were then lysed and FLAG-tagged surface proteins precipitated with protein G-Sepharose beads. Subsequently the proteins were boiled and ran in SDS-Page gels under denaturating and reducing conditions. H antigenic materials were then detected using an anti-H polyclonal antibody. Of note, determination of hetero-oligomeric complexes could be determined only between headless and standard H proteins because of their clear mobility shifts in SDS-Page (lines 6 and 9). (B) Syncytium formation assay. Vero-cSLAM cells were transfected with an F-expressing vector and a plasmid encoding a CDV H double mutant (H-N128D/F131Y) that lacked mAb αH-1347 binding activity. Four hours post-transfection, cells were treated, or not, with mAb αH-1347 (1:500). Representative fields of view of cell-cell fusion induced 24h post-transfection are shown. (C) Reactivity of standard and N128D/F131Y H mutant to mAbs αH-1347 and αFLAG recorded by flow cytometry analyses after addition of the secondary antibody. Means ± S.D. of data from three independent experiments performed in triplicates are shown.
(TIF)
