# Supplementary figures and images for: Sequential Conformational Changes in the Morbillivirus Attachment Protein Initiate the Membrane Fusion Process
Source: PLoS Pathog. 2015 May 6;11(5):e1004880. doi: 10.1371/journal.ppat.1004880 (PMC4422687; doi:10.1371/journal.ppat.1004880)

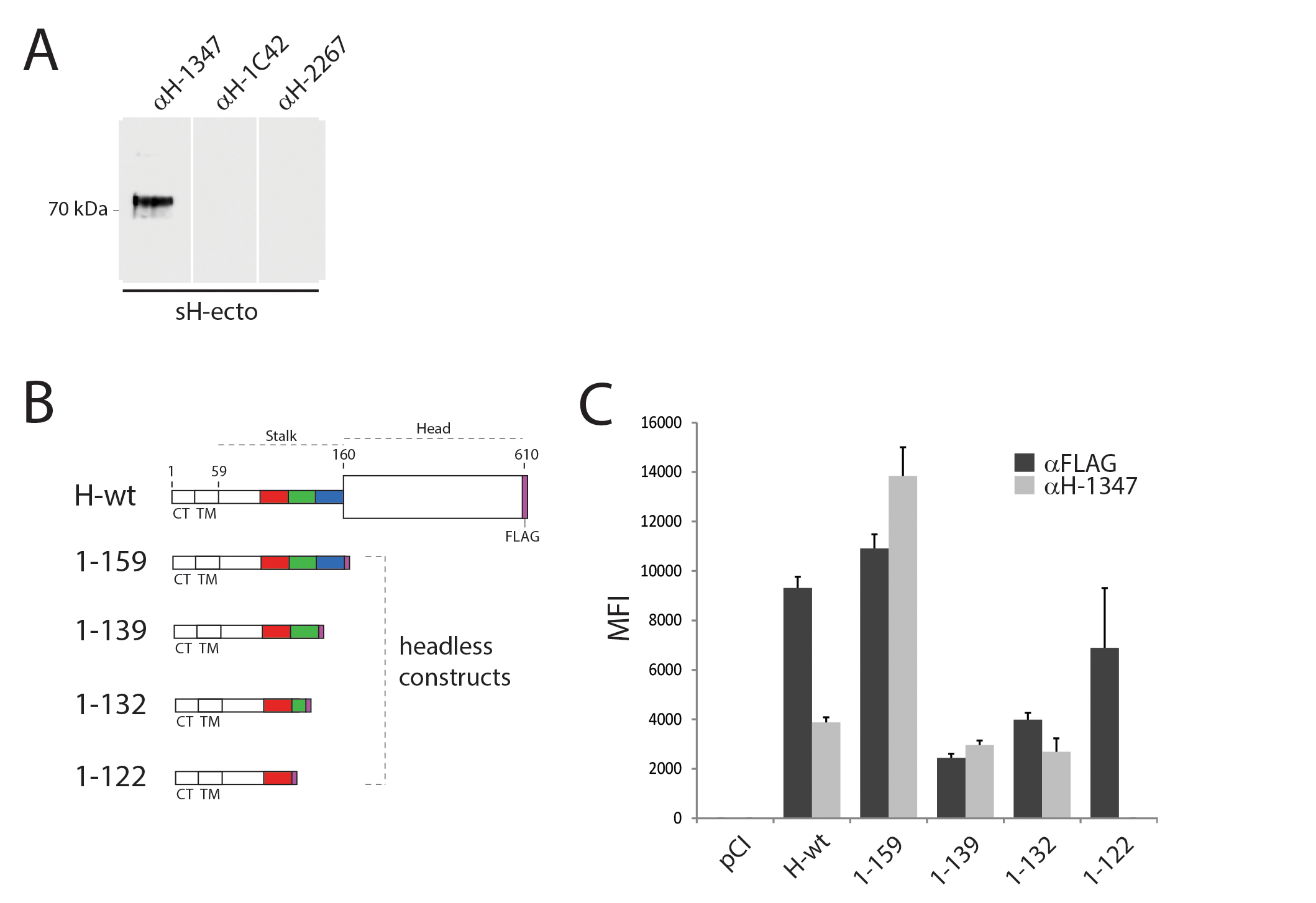

Supplement: S1 Fig — (A) The sol H-ecto construct was expressed three days in 293T cells. Antigenic materials from harvested supernatants were run in an 8% SDS-Page gel under reducing conditions and subsequently detected using different monoclonal antibodies (as indicated in the text). (B) Schematic representation of the full length CDV H protein with the main functional domains in the stalk region color-coded. The length and the functional domain(s) deleted of each headless H variants are represented below. (C) The wt H protein and respective heads-deleted versions were expressed one day in Vero cells. Reactivity of standard and derivate headless H mutants to mAb αH-1347 and αFLAG were then determined by flow cytometry analyses after addition of the secondary antibody. Means ± S.D. of data from three independent experiments performed in triplicates are shown. (TIF) [file ppat.1004880.s001.tif]

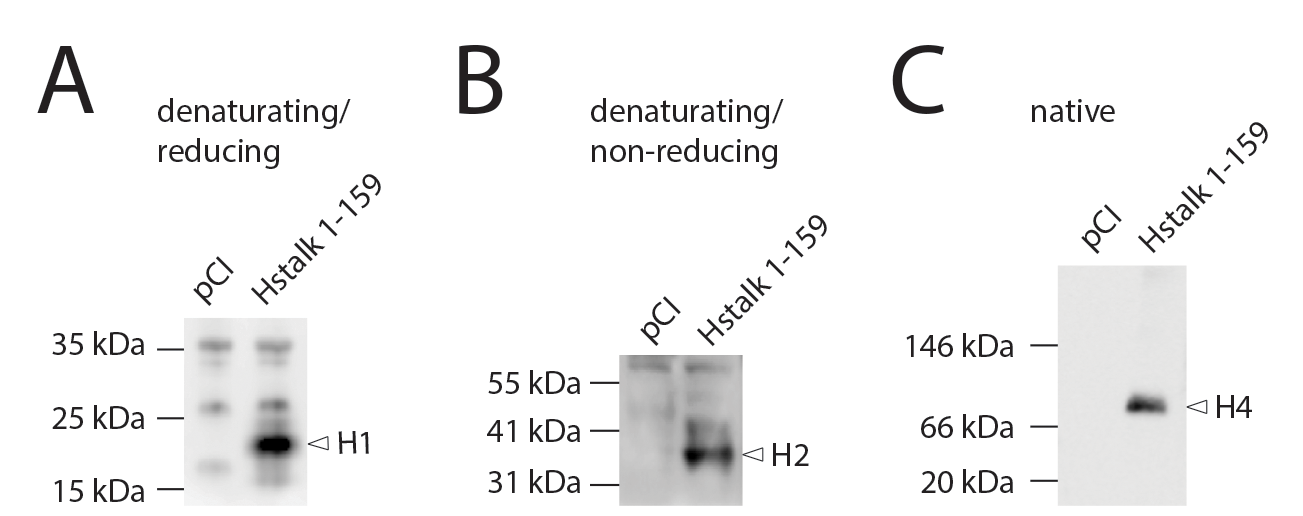

Supplement: S2 Fig — (A) The H construct was expressed one day in Vero cells and its oligomeric state subsequently investigated by immunoblotting using an anti-H polyclonal antibody of gels ran under (A) denaturating and reducing conditions, (B) denaturating and non-reducing conditions and (C) native conditions. The molecular weight of the markers is shown on the left side of each gel. H1: monomeric H; H2: dimeric H; H4: tetrameric H. (TIF) [file ppat.1004880.s002.tif]

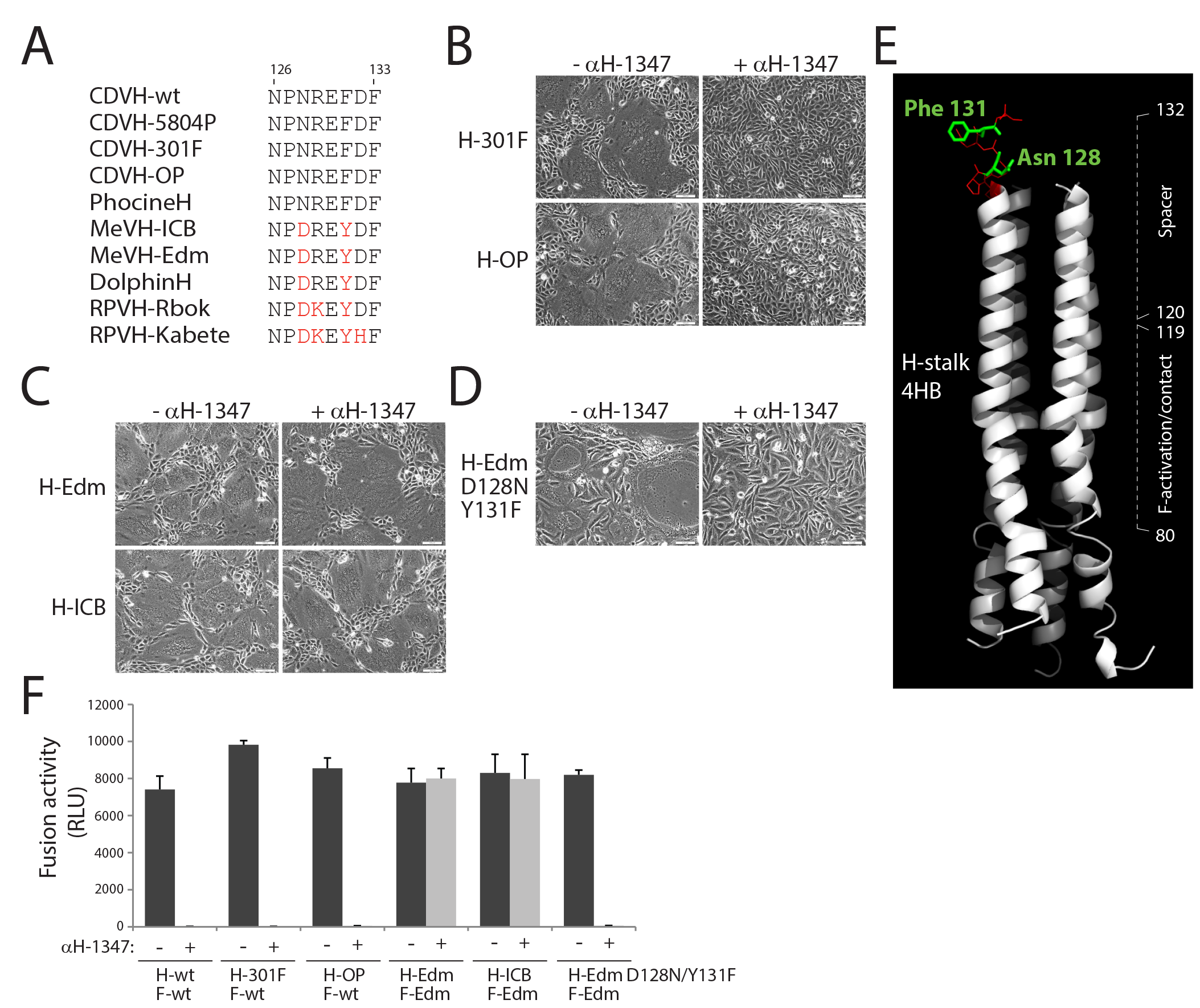

Supplement: S3 Fig — (A) Sequence alignment of the mAb αH-1347 epitope of various members of the morbillivirus genus. (B, C and D) Syncytium formation assay. Vero-cSLAM cells were transfected with the indicated H-expressing plasmids together with the indicated F-expressing plasmid in the presence or absence of mAb αH-1347. Representative fields of view of cell-cell fusion induced 24h post-transfection are shown. (E) Homology structural model of the 4-helical bundle CDV H-stalk region [31]. The atomic coordinates of the visible residues involved in mAb αH-1347 epitope are color-coded in red. In addition, the two residues that were shown to strongly influence mAb αH-1347 binding activity are highlighted in green. (F) Quantitative fusion assay. The fusion promotion efficiency of each H/F combinations (in the presence or absence of the mAb) was determined as described in the legend of Fig 1B. Means ± S.D. of data from three independent experiments performed in triplicates are shown. (TIF) [file ppat.1004880.s003.tif]

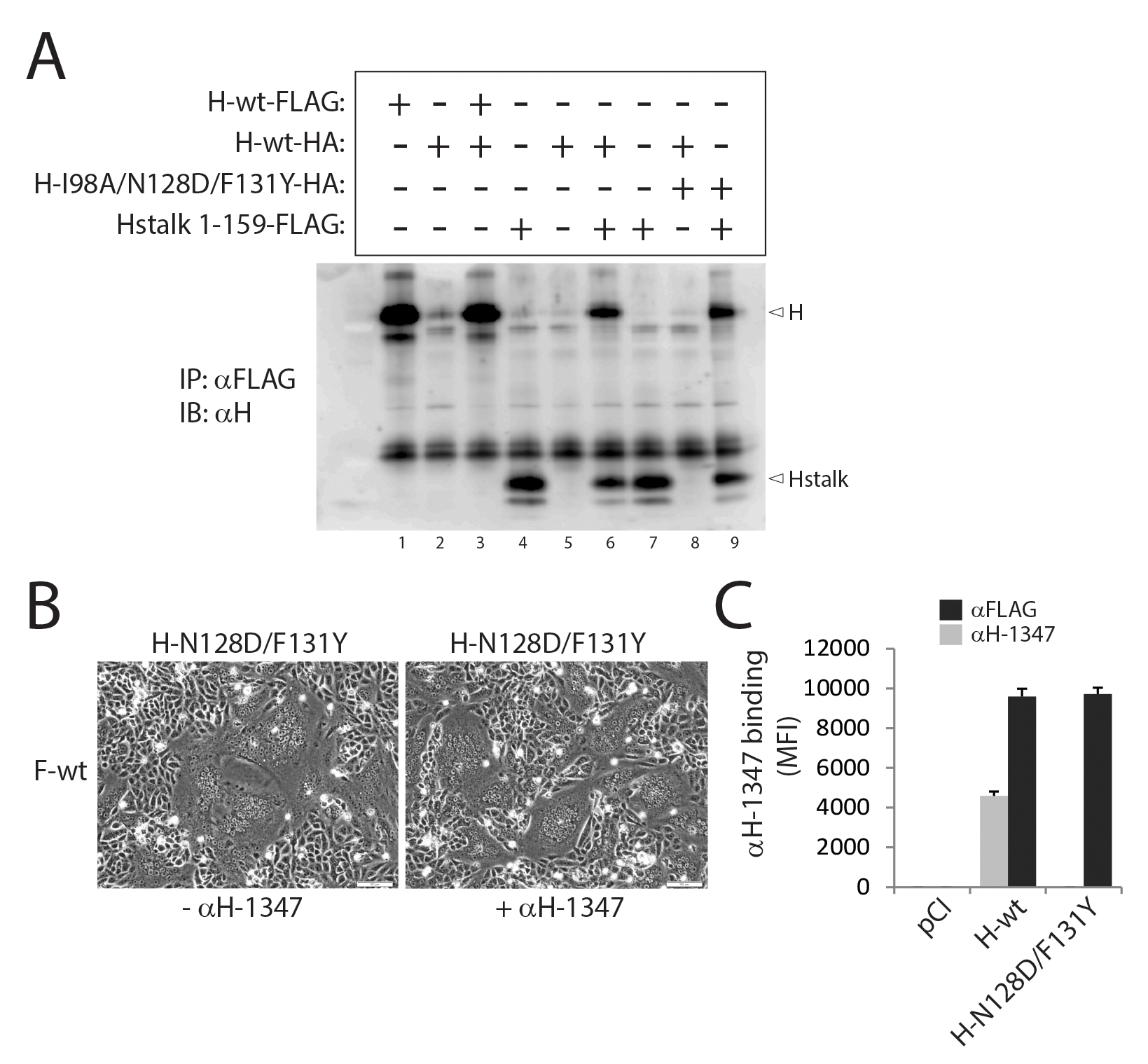

Supplement: S4 Fig — (A) Cell surface co-immunoprecipitation experiments were performed to assess the formation of H hetero-oligomeric complexes. HA or FLAG-tagged H variants were expressed alone, or in combination, in Vero cells (as indicated in the upper panel). One day post-transfection, Vero cells were decorated with anti-FLAG mAb for 1 hour at 4°C. Cells were then lysed and FLAG-tagged surface proteins precipitated with protein G-Sepharose beads. Subsequently the proteins were boiled and ran in SDS-Page gels under denaturating and reducing conditions. H antigenic materials were then detected using an anti-H polyclonal antibody. Of note, determination of hetero-oligomeric complexes could be determined only between headless and standard H proteins because of their clear mobility shifts in SDS-Page (lines 6 and 9). (B) Syncytium formation assay. Vero-cSLAM cells were transfected with an F-expressing vector and a plasmid encoding a CDV H double mutant (H-N128D/F131Y) that lacked mAb αH-1347 binding activity. Four hours post-transfection, cells were treated, or not, with mAb αH-1347 (1:500). Representative fields of view of cell-cell fusion induced 24h post-transfection are shown. (C) Reactivity of standard and N128D/F131Y H mutant to mAbs αH-1347 and αFLAG recorded by flow cytometry analyses after addition of the secondary antibody. Means ± S.D. of data from three independent experiments performed in triplicates are shown. (TIF) [file ppat.1004880.s004.tif]
